# Multi-model evaluation on integrated quality regionalization of the medicinal and edible plant *Smilax glabra*

**DOI:** 10.3389/fpls.2026.1966447

**Published:** 2026-10-14

**Authors:** Bin Huang, Heng Jiang, Huiming Wang, Shengni Liu, Ting Xie, Baojing Li, Ting Chen

**Affiliations:** 1 Sino-Pakistan International Center on Traditional Chinese Medicine, School of Pharmaceutical Sciences, Hunan Medical College, Huaihua, China; 2 Yunnan Key Laboratory of Southern Medicinal Utilization, College of Traditional Chinese Medicine, Yunnan University of Chinese Medicine, Kunming, Yunnan, China; 3 Key Laboratory of State Administration of Traditional Chinese Medicine for Production & Development of Cantonese Medicinal Materials, Guangzhou Comprehensive Experimental Station of National Industrial Technology System for Chinese Materia Medica, Guangdong Engineering Research Center of Good Agricultural Practice & Comprehensive Development for Cantonese Medicinal Materials, School of Chinese Materia Medica, Guangdong Pharmaceutical University, Guangzhou, China

**Keywords:** ArcGIS, co-kriging interpolation, integrated quality regions, MaxEnt, multi-model evaluation, *Smilax glabra*

## Abstract

Under climate change, shifts in the geographic distribution of medicinal plants and the mechanisms underlying their quality responses provide the scientific basis for forward-looking conservation and utilization strategies. *Smilax glabra* Roxb., an important medicinal and edible plant in China, is currently threatened by both habitat degradation and rising market demand, yet systematic research on the evolution of its suitable habitat and the spatial patterns of quality formation remains limited. Using 571 spatially rarefied occurrence records, this study integrated ten species distribution algorithms—including an ENMeval-optimized MaxEnt model, generalized linear models, and several machine learning methods—with environmental variables screened by Spearman correlation (with FDR correction) and variance inflation factor analysis. An ensemble forecasting framework was constructed on the Biomod2 platform to simulate habitat suitability for *S. glabra* under current conditions and four shared socioeconomic pathways (SSP126, SSP245, SSP370, and SSP585) for the 2050s and 2090s. The results showed that the dominant factors limiting its distribution were precipitation of the driest month (Bio14, 56% contribution), annual precipitation (Bio12, 28.9%), and minimum temperature of the coldest month (Bio6, 7.2%), with suitable ranges of 15.36~217.80 mm, 1194.92~4222.90 mm, and –0.34~23.11 °C, respectively. Future projections indicated an overall southwestward shift of the suitable habitat centroid. Stepwise movement analysis revealed that the centroid shifted markedly as early as the 2050s under climate change, with a displacement exceeding 625 km relative to the present under SSP245, and a migration distance exceeding 450 km by the 2090s under SSP585. Area statistics further showed that highly suitable habitat did not contract under the high-emission scenario but instead expanded locally. Astilbin contents from 30 geographic populations were then used to generate a chemical suitability layer via universal co-kriging with leave-one-out cross-validation and kernel density uncertainty assessment. After rigorously excluding areas at high risk of spatial extrapolation, this layer was overlaid with the ecological suitability layer to delineate, for the first time, an Integrated quality regions for *S. glabra*. The reliable core Integrated quality regions was concentrated in southwestern Guizhou and northern and southeastern Guangxi. The highly quantitative, statistically bias-avoiding, multi-model two-dimensional assessment framework developed here overcomes the limitations of single-suitability evaluations and provides spatially explicit theoretical support for germplasm conservation, habitat-approximation cultivation site selection, and climate-adaptive management of *S. glabra*.

## 1 Introduction

*Smilax glabra* Roxb. is a perennial climbing plant belonging to the family Smilacaceae. Its dried rhizome is known as Tufuling, a traditional Chinese medicinal material, which is widely distributed across several provinces in southern China (Guo et al., 2024). This medicinal herb is first recorded in the *Bencaojing Jizhu* during the Southern and Northern Dynasties and is characterized by its sweet, bland, and neutral properties. It is associated with the liver and stomach meridians and is reputed to have detoxifying, damp-dispelling, and joint-relieving effects, making it clinically useful for conditions such as damp-heat bi syndrome and carbuncles (Wu et al., 2022). Modern research has confirmed that *S. glabra* is rich in various bioactive components, including flavonoids (such as astilbin), phenylpropanoids, and steroids (Hua et al., 2018; Li et al., 2026b). Astilbin, in particular, has been identified as a key quality marker and is utilized for the authentication and quality assessment of the herb (Shi et al., 2023). Pharmacological studies indicate that its extracts and isolated compounds exhibit multiple biological activities, including anti-inflammatory, antioxidant, immunomodulatory, uric acid-lowering, and hepatoprotective effects (Wang et al., 2019; Tian et al., 2025; Wei et al., 2025; Guo et al., 2026), demonstrating potential applications in the intervention of hyperuricemia, heavy metal poisoning, and metabolic diseases. Additionally, *S. glabra* is also valued as a dual-purpose resource in food and medicine, finding extensive use in the development of functional foods such as turtle jelly and health teas (Shi et al., 2023; Wu et al., 2026). Sustainable utilization of this resource is critical for ensuring the quality of medicinal materials and the healthy development of the industry.

However, the current *S. glabra* industry is facing a severe resource crisis and market disorder. Because of its long growth cycle and the traditional harvesting practice of uprooting the entire plant, long-term predatory overharvesting has led to a sharp decline in wild populations and severe habitat destruction (Li et al., 2002; Liang et al., 2025). With the continuous surge in demand from the medicinal and health care markets, domestic wild resources can no longer support industrial development, and large quantities must be imported from Southeast Asian countries to fill the supply gap (Liang et al., 2025). Resource scarcity has further exacerbated market disorder, leading to the influx of numerous closely related plants from the genera *Smilax* and *Heterosmilax* (such as *Heterosmilax japonica*) as substitutes or adulterants, seriously threatening the safety and efficacy of clinical medication (Li et al., 2001, 2002). To alleviate the pressure of dwindling wild resources and ensure the authenticity of medicinal materials, the development of standardized artificial cultivation, such as understory intercropping, has become an inevitable choice for the industry (Liang et al., 2025; Liang and Zhang, 2026). Although artificial cultivation is key to breaking the deadlock, existing work still has obvious limitations: current research mostly focuses on chemical composition analysis, pharmacological mechanisms, and quality control technologies (Jeeno et al., 2022; Shi et al., 2023), while among the more than one hundred compounds identified, integrated research on cultivation ecology and quality formation mechanisms is seriously lacking; the interaction network among environmental factors, plant physiology, and secondary metabolites has not been elucidated, resulting in spatial blindness in the selection of artificial introduction sites. In addition, as a vine highly dependent on a warm and humid climate, *S. glabra* is extremely sensitive to global warming and extreme hydrothermal conditions, making it urgent to assess its future spatial trajectory to guide proactive conservation. Traditional single ecological suitability prediction can no longer meet the demands of high-quality medicinal material production, and the coordinated evaluation of quality suitability and ecological suitability is becoming a new paradigm for traditional Chinese medicinal material zoning (Tavan et al., 2025; Zhou et al., 2025), aiming to identify Integrated quality regions that balance biomass accumulation and active ingredient synthesis (Jiang et al., 2026a), providing a scientific basis for optimizing genuine producing areas, selecting sites for analogous habitat cultivation, and sustainably conserving resources.

Species distribution models (SDMs) are essential tools for analyzing habitat changes of medicinal plants driven by climate factors. The MaxEnt model is widely used due to its robustness with small sample sizes, and when combined with parameter optimization through ENMeval, it effectively reduces the risk of overfitting (Muscarella et al., 2014). Integrated modeling platforms such as Biomod2 leverage the advantages of multiple algorithms, including GLM, GAM, BRT, and RF, significantly enhancing predictive reliability and spatial extrapolation robustness (Thuiller, 2003; Zhang et al., 2025). Ecological niche models, by coupling species occurrence records with environmental variables such as climate, soil, and topography, can accurately identify key driving factors and quantify their contributions (Li et al., 2025a; Xiang et al., 2025). However, single models are susceptible to algorithmic bias, and employing a multi-model integration strategy can enhance the credibility of the results (Buisson et al., 2010; Guo et al., 2025a).

This study focuses on *S. glabra*, integrating its effective geographical distribution records with selected multi-source environmental variables (climate, topography, soil). Utilizing the Biomod2 platform, we incorporated nine algorithms along with an optimized MaxEnt model to predict the dynamics of potential suitable habitats under current and future climate scenarios (SSP126, SSP245, SSP370, and SSP585). Concurrently, we collected data on the content of key components, astilbin, from 30 representative populations, and constructed a quality suitability distribution map using co-kriging interpolation. Furthermore, we overlaid ecological and quality suitability layers to delineate, for the first time, the Integrated quality regions of *S. glabra*. The objectives of this research are to: (1) analyze the key environmental variables driving its distribution; (2) assess trends in future habitat suitability changes; and (3) reveal the spatial coupling relationship between climate and quality. This study aims to provide theoretical support and practical pathways for the conservation of *S. glabra* genetic resources, planning of high-quality production areas, and adaptive cultivation in response to climate change.

## 2 Materials and methods

### 2.1 Species data collection and processing

Geographical distribution records for *Smilax glabra* were obtained from the Chinese Virtual Herbarium (CVH, http://www.cvh.ac.cn), the National Specimen Information Infrastructure (NSII, http://www.nsii.org.cn/2017/home.php), and the Global Biodiversity Information Facility (GBIF, https://www.gbif.org/). After removing duplicates and correcting coordinates, a total of 672 independent records containing precise latitude and longitude were obtained. To minimize spatial sampling bias and autocorrelation effects on the models, the Spatially Rarefy Occurrence Data tool from the SDM Toolbox was utilized, with a distance threshold set at 5 km (Huang et al., 2024). This process resulted in the retention of 571 high-confidence distribution records for modeling (**Figure 1**). It is important to note that the aforementioned data primarily consist of specimen records and do not distinguish between wild and cultivated individuals; thus, the modeling dataset may include both. Due to the difficulty in tracing the cultivation history of each point and the fact that traditional cultivation often manifests as semi-wild management in suitable habitats, the ecological requirements of cultivated individuals may overlap with those of wild populations. Therefore, potential cultivated points were not actively excluded. This study aims to predict the potential ecological suitability of the species and provide a basis for site selection in simulated cultivation.

**Figure 1:**
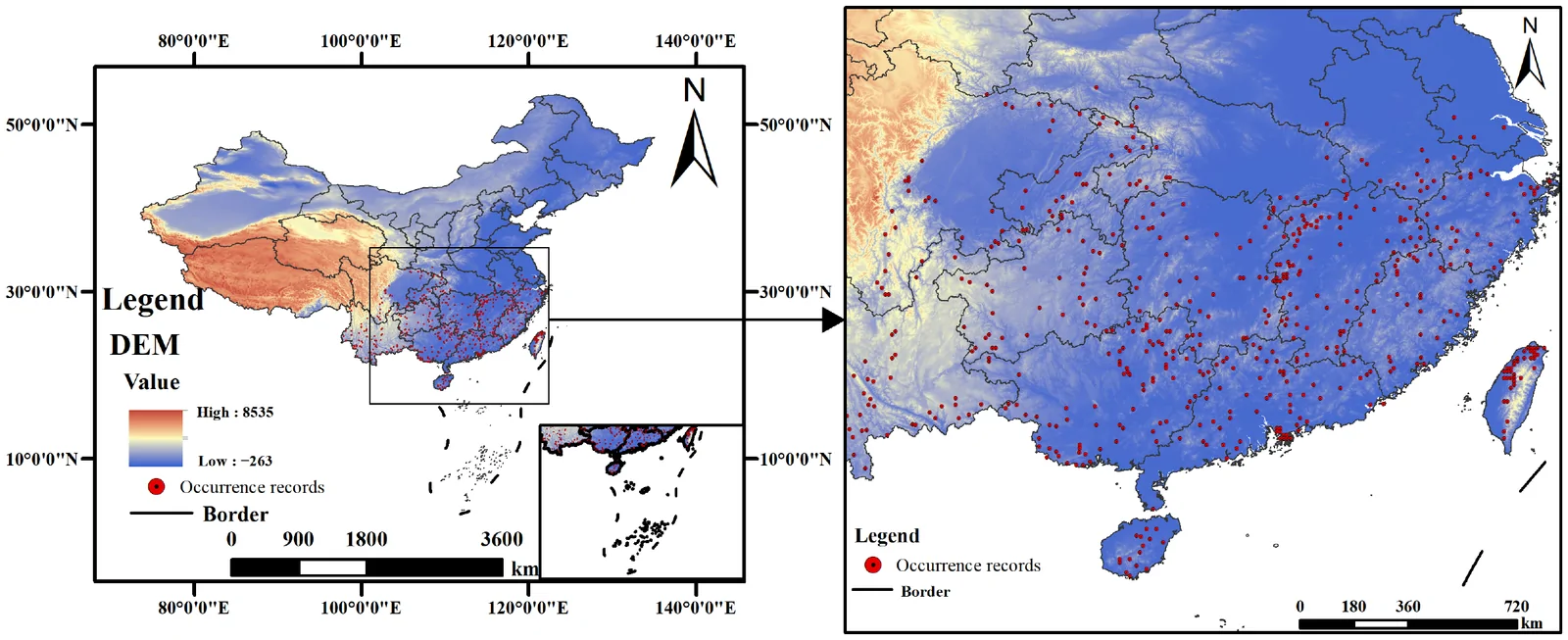
The occurrence records of *Smilax glabra*.

To mitigate inherent biases in specimen data collection (e.g., concentration in easily accessible areas), several measures were implemented during model construction: First, a substantial number of pseudo-absence points were randomly sampled from the background environment across the study area in China to explore the environmental space of unsampled regions, thereby reducing accessibility bias. Second, an ensemble approach utilizing multiple algorithms, such as Random Forest and Generalized Additive Models, was employed to leverage the differing responses of algorithms to biases, thereby suppressing overfitting associated with any single model and enhancing prediction stability. Third, environmental variable contribution assessments and correlation screenings were conducted to ensure that selected factors have clear ecological significance rather than being solely related to biased sampling patterns. These comprehensive strategies allowed for the development of models grounded in more universal environmental relationships, thereby more reliably revealing the potential distribution and quality formation patterns of *S. glabra*.

### 2.2 Selection of environmental variables and climate scenario setting

To comprehensively characterize the habitat features of *S. glabra*, this study selected three categories of environmental variables: bioclimatic factors, soil factors, and topographic factors. Nineteen bioclimatic variables representing current (1970~2000) and future periods were obtained from the WorldClim database (http://www.worldclim.org/), with a spatial resolution of 2.5 arc minutes. The BCC-CSM2-MR global climate model was utilized as the primary data source for future climate predictions, demonstrating strong performance in simulating extreme temperatures and precipitation in China. Based on the CMIP6 framework, four Shared Socioeconomic Pathways (SSPs) were selected to encompass different radiative forcing scenarios: SSP126 (sustainable development), SSP245 (intermediate pathway), SSP370 (regional competition), and SSP585 (fossil fuel-driven) (Li et al., 2023). The prediction periods were set for the 2050s (2041~2060) and 2090s (2081~2100). Soil data were sourced from the Harmonized World Soil Database (https://www.fao.org/soils-portal/data-hub/soil-maps-and-databases/harmonized-world-soil-database-v12/en/), while topographic data were obtained from WorldClim (http://www.worldclim.org/). All environmental layers were cropped to the extent of China.

To reduce multicollinearity among the variables (**Table 1**), a two-step variable selection process was employed. First, based on the initial results from the MaxEnt model, variables with zero contribution rates were eliminated. Second, Pearson correlation analysis and Variance Inflation Factor (VIF) calculations were performed on the remaining variables. For highly correlated pairs (|r| > 0.8), known physiological traits of *S. glabra* were taken into account, and factors with clearer ecological significance and higher contribution rates in the preliminary model were preferentially retained. This screening strategy, based on contribution rates and correlation, represents an established practice for enhancing model performance and interpretability (Zhang et al., 2011). Iterative screening continued until the VIF values of all retained variables fell below 10, thereby ensuring variable independence while preserving the most ecologically informative predictors and constructing a robust habitat model (Pradhan, 2016).

**Table 1:** All environmental variables.

Variables	Name	Unit
Bio1	Annual mean temperature	°C
Bio2	Mean diurnal temperature range	°C
Bio3	Isothermality	/
Bio4	Temperature seasonality	/
Bio5	Maximum temperature of the warmest month	°C
Bio6	Minimum temperature of the coldest month	°C
Bio7	Temperature annual range (Bio5−Bio6)	°C
Bio8	Mean temperature of the wettest quarter	°C
Bio9	Mean temperature of the driest quarter	°C
Bio10	Mean temperature of the warmest quarter	°C
Bio11	Mean temperature of the coldest quarter	°C
Bio12	Annual precipitation	mm
Bio13	Precipitation of the wettest month	mm
Bio14	Precipitation of the driest month	mm
Bio15	Precipitation seasonality	/
Bio16	Precipitation of the wettest quarter	mm
Bio17	Precipitation of the driest quarter	mm
Bio18	Precipitation of the warmest quarter	mm
Bio19	Precipitation of the coldest quarter	mm
Awc_class	Soil available water content	%
Slope	Slope	%
Elev	Elevation	m
Aspect	Aspect	/
T_ph_h2o	Topsoil pH	-log(H+)
S_ph_h2o	Subsoil pH	-log(H+)
T_oc	Topsoil organic carbon content	%weight
S_oc	Subsoil organic carbon content	%weight
T_clay	Topsoil clay content	%weight
S_clay	Subsoil clay content	%weight
T_sand	Topsoil sand content	%weight
S_sand	Subsoil sand content	%weight
T_silt	Topsoil silt content	%weight
S_silt	Subsoil silt content	%weight
T_ece	Topsoil electrical conductivity	ds/m
S_ece	Subsoil electrical conductivity	ds/m
T_caco3	Topsoil carbonate or lime content	%weight
S_caco3	Subsoil carbonate or lime content	%weight

### 2.3 Model construction, optimization, and evaluation

The potential distribution of *S. glabra* was simulated using MaxEnt 4.4.1. Both the modeling and subsequent analyses relied on the environmental variables selected in Section 2.2. To improve model generalization and reduce overfitting, the ENMeval package in R was used to systematically tune the regularization multiplier (ranging from 0.5 to 4.0 at intervals of 0.5) and feature class combinations (L, LQ, H, LQH, LQHP, LQHPT) (Pradhan, 2016; Zhao et al., 2021b). The optimal parameter set was determined by minimizing the corrected Akaike Information Criterion, and this tuning process was performed on the already filtered, non-redundant variable set. Basic model settings were as follows: 75% of occurrence points were randomly assigned to the training set and 25% to the test set; the maximum number of iterations was set to 1,000,000 (actual convergence was controlled by the default threshold of 1.0×10^-5^). A 10-fold cross-validation and the jackknife method were applied to assess variable contributions. Model performance was evaluated using the area under the receiver operating characteristic curve (AUC), where an AUC ≥ 0.8 indicated reliable predictions and a value between 0.9 and 1.0 denoted high accuracy (Huang and Ling, 2005). To ensure methodological consistency, the true skill statistic (TSS) and Kappa statistic were also calculated alongside AUC for joint evaluation of the MaxEnt and Biomod2 models. Using the optimized model, potential suitable habitats were predicted under current conditions and for two future periods (2050s and 2090s) across four Shared Socioeconomic Pathways (SSP126, SSP245, SSP370, SSP585).

For all suitability distribution maps and subsequent spatial classifications of the integrated quality index, the natural breaks method was applied consistently. This approach allows objective classification based on the inherent distribution characteristics of each raster dataset, avoiding subjective threshold bias and ensuring that categories such as highly suitable areas reflect the natural data structure within each spatiotemporal scenario. When making cross-scenario comparisons, the same classification algorithm ensures that the analysis focuses on spatial dynamics (e.g., centroid shifts and changes in suitable area) of “relatively high-suitability areas,” thereby guaranteeing that observed trends are driven by climate change rather than by differences in classification methods (Harrigan, 2025).

To assess prediction uncertainty and enhance model robustness, a multi-model ensemble framework was constructed. Nine algorithms were initially considered: Generalized Linear Models (GLM), Generalized Additive Models (GAM), Gradient Boosting Machines (GBM), Classification Tree Analysis (CTA), Artificial Neural Networks (ANN), Surface Range Envelopes (SRE), Flexible Discriminant Analysis (FDA), Multivariate Adaptive Regression Splines (MARS), and Random Forest (RF). These algorithms were implemented using the Biomod2 package in R Studio 4.4.1 (Zhao et al., 2021a), with 10 repetitions each, a 75% training/25% validation split, and 1,000 pseudo-absences generated from the previously screened effective occurrence records to strictly maintain class balance for the machine learning algorithms. Model performance was assessed using the TSS, the Kappa coefficient, and the AUC, with inclusion criteria set as AUC ≥ 0.80, TSS ≥ 0.60, and Kappa ≥ 0.60 (Allouche et al., 2006). The final ensemble prediction was generated through a two-step procedure: first, Biomod2 models meeting all three thresholds were selected; second, their predictions were combined with that of the independently optimized MaxEnt model to produce a consensus ensemble prediction. For habitat suitability classification, to address the threshold drift problem in cross-scenario comparisons, the traditional natural breaks method was abandoned. Instead, the maximum sensitivity plus specificity (Max SSS) threshold derived from the current-period ensemble model was extracted as a fixed absolute biological benchmark and strictly applied to reclassify probability rasters across all periods and scenarios. By comparing suitable habitat areas, spatial pattern dynamics, and continuous suitability probability differences (Delta Maps) among scenarios and periods, the potential impacts of climate change on the distribution of *S. glabra* were elucidated.

### 2.4 Spatial interpolation analysis of astilbin content

To characterize the geographical variation in medicinal quality of *S. glabra*, astilbin—the marker compound specified in the 2025 edition of the Chinese Pharmacopoeia—was selected as the chemical indicator (Zhong et al., 2026). A systematic search of CNKI (https://www.cnki.net) and Google Scholar (https://scholar.google.com) was conducted for literature published between 2005 and 2025 (search date: 2 January 2026), yielding 30 astilbin content records with precise geographic coordinates. To ensure high comparability and reliability of data compiled from different sources, strict literature screening criteria were established. All included records employed standardized ultrasonic extraction protocols and high-performance liquid chromatography (HPLC) for quantification. The original source, specific extraction method, analytical instrument parameters, and unified units (mg/g) for each record are documented in detail in **Supplementary Table 1**, providing a solid data foundation for subsequent spatial interpolation analysis. Although the sample size was limited, the records covered the core genuine producing regions, thereby capturing quality differences along major environmental gradients. Spearman’s rank correlation analysis was performed between astilbin content values and the initial 37 environmental variables. This non-parametric method does not require linear relationships or normal distribution and is therefore suitable for exploring monotonic associations between secondary metabolites and environmental factors. To effectively control the false-positive rate arising from multiple statistical tests, Benjamini-Hochberg FDR correction was applied to the P values of the correlation analysis (**Supplementary Figure 1**).

Using the key environmental variables that remained significantly correlated after correction as covariates, universal co-kriging interpolation was performed in the Geostatistical Analyst module of ArcGIS 10.8. During model fitting, the content data were first log-transformed, and a second-order local polynomial was applied to remove global trend. The empirical semivariogram was then fitted with a stable model (parameter = 2), with anisotropy set to false. The lag structure for spatial autocorrelation was optimized to 12 lags with a lag size of 0.766. The search neighborhood for interpolation followed a standard four-sector design (45° offset), with a maximum of 5 and a minimum of 2 neighbors per sector, and both major and minor search radii set to 4.62. To rigorously evaluate the predictive accuracy and reliability of the interpolation model, leave-one-out cross-validation (LOOCV) was employed. Validation results yielded a mean error (ME) of -1.57, a root mean square error (RMSE) of 12.74, a mean standardized error (MSE) of -0.116, and a root mean square standardized error (RMSSE) of 0.504 (**Supplementary Figure 1**). The near-zero ME and MSE indicate an overall unbiased interpolation model, while the reasonable RMSE and RMSSE further confirm the validity of the spatial interpolation results. In addition, given the spatial limitations of the chemical indicator samples, kernel density estimation (KDE) was used to generate a spatial density map of sampling points (**Supplementary Figure 2**) to avoid misleading conclusions caused by excessive spatial extrapolation, and a prediction standard error (PSE) map was simultaneously produced during co-kriging interpolation. Through these uncertainty quantification approaches, areas lacking empirical data support were explicitly delineated and marked in the final integrated zoning map, thereby ensuring rigor and scientific validity in analyzing the environment–quality coupling relationship.

### 2.5 Centroid migration

Using the Mean Center tool within the spatial statistics module of ArcGIS 10.8 (Regos et al., 2019), we calculated the geometric centroids of suitable habitats for *S. glabra* under different time periods (present, 2050s, and 2090s) and climate scenarios. This analysis quantified the direction and distance of habitat shifts in geographical space, revealing the response trends and spatial migration patterns of *S. glabra* in relation to future climate change.

### 2.6 Delineation of integrated quality regions

To identify production-potential areas that combine high ecological adaptability with superior medicinal quality, an integrated quality assessment system coupling ecological and chemical suitability was developed. This system integrates an ensemble model-derived ecological suitability probability raster, representing survival potential, with a co-kriging interpolated astilbin content raster, representing chemical quality. A weighted overlay approach was used to calculate the integrated quality index (IQI). Under the baseline scenario, the weights for ecological suitability and chemical quality suitability were each set to 0.5. The rationale for this equal-weight scheme is that, in ecological–chemical zoning studies of medicinal plants, survival potential and medicinal value are regarded as two equally fundamental dimensions defining geo-authenticity and sustainable resource utilization; in the absence of precisely quantified *a priori* contributions, equal weighting is transparent, neutral, and widely adopted in this field (Zhang et al., 2025). Finally, the natural breaks method was applied to classify the IQI under the baseline weights, generating integrated quality zones of different grades to precisely identify key areas where ecological and quality suitability are jointly optimized (Jiang et al., 2025), thereby providing a scientific basis for defining the geo-authenticity of *S. glabra* and for cultivation planning. In addition, to verify the robustness of the weight assignment scheme, a sensitivity analysis was conducted using alternative weight combinations (0.3:0.7 and 0.7:0.3), ensuring that the spatial delineation of Integrated quality regions would not shift substantially in response to minor weight adjustments.

## 3 Results

### 3.1 Model parameter optimization and performance evaluation

Using the environmental variables selected in Section 2.2, the ENMeval package was employed to systematically tune the regularization multiplier (RM) and feature class (FC) combinations for the MaxEnt model. The results showed that the parameter combination RM = 2 and FC = LQH achieved the best balance between model complexity and goodness-of-fit, with a Delta AICc value of 0.00, significantly outperforming other combinations (**Figure 2**). Compared with the default parameters (RM = 1, FC = LQHPT), the optimized model maintained a mean validation AUC of 0.894, reduced the mean AUC difference to 0.042, and lowered the 10% training omission rate to 0.138, indicating good discriminative ability and stability. In addition, the number of model coefficients decreased from 63 (default) to 22, and the gap between training and validation AUC narrowed, effectively controlling overfitting risk. Therefore, RM = 2 and FC = LQH were adopted as the optimal parameter set for subsequent analyses. The predictive performance of the nine candidate algorithms was assessed (**Supplementary Figure 3**; **Table 2**). A more stringent set of model screening thresholds was applied (AUC ≥ 0.80, TSS ≥ 0.60, Kappa ≥ 0.60). GBM, FDA, GAM, MARS, and CTA all met these predefined criteria and were therefore retained for ensemble modeling, indicating their capacity to effectively capture the distribution patterns of *S. glabra*.

**Figure 2:**
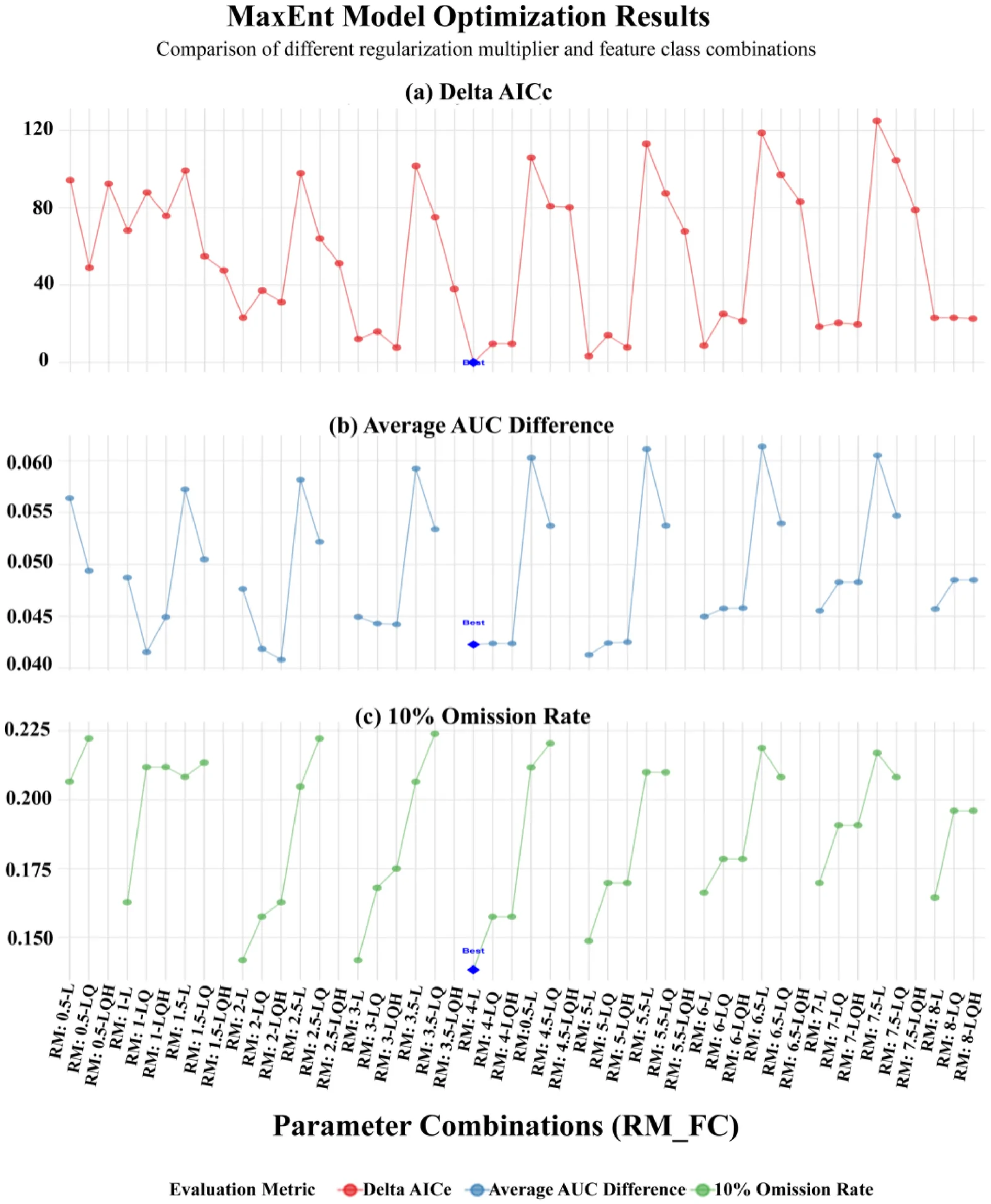
Optimization of the MaxEnt model: **(A)** delta AICc; **(B)** average AUC difference; **(C)** 10% omission rate.

**Table 2:** Performance metrics (AUC, TSS, and kappa) of the nine species distribution models evaluated for predicting the potential geographic distribution of *Smilax glabra*.

Indicator	Algorithm								
	GBM	FDA	GAM	MARS	RF	CTA	ANN	GLM	SRE
AUC	0.8902	0.8902	0.8880	0.8868	0.8832	0.8349	0.8062	0.7803	0.6641
TSS	0.6989	0.6900	0.6808	0.6946	0.6319	0.6606	0.6002	0.5601	0.3282
Kappa	0.6390	0.6366	0.6278	0.6339	0.5797	0.6098	0.5477	0.5316	0.3295

### 3.2 Identification of dominant environmental factors and suitability threshold analysis

Through an initial screening of variable contributions, Pearson correlation analysis (**Supplementary Figure 4**), and variance inflation factor testing, 14 key environmental variables were selected from the initial set of 37 for final model construction. The optimized MaxEnt model (RM = 2, FC = LQH) achieved a mean test AUC of 0.906, demonstrating good discriminative power. Moreover, the TSS and Kappa values derived from the model output both reached 0.763, demonstrating that the optimized model has high predictive accuracy and excellent discriminative capacity for the potential distribution of *S. glabra*. Variable contribution rates (**Table 3**) showed that precipitation of the driest month (Bio14) ranked highest (56%), followed by annual precipitation (Bio12, 28.9%), minimum temperature of the coldest month (Bio6, 7.2%), and subsoil organic carbon content (S_oc, 3.1%). The top four variables together contributed 95.2% and were identified as the dominant factors limiting the distribution of *S. glabra*. The remaining variables (slope, temperature annual range, subsoil clay content, etc.) each contributed less than 3%. Response curves (**Figure 3**) and suitability thresholds (presence probability > 0.5, **Table 4**) indicated that *S. glabra* depends on warm and humid climatic conditions. In summary, the distribution of *S. glabra* is primarily constrained by the dual stresses of low temperature and seasonal drought, with its core areas showing strong spatial correspondence to subtropical humid monsoon climates.

**Table 3:** Importance of key environmental factors affecting the distribution of *Smilax glabra* in China, as derived from the optimized MaxEnt model.

Variables	Name	Percent contribution (%)
Bio14	Precipitation of the driest month	56.0
Bio12	Annual precipitation	28.9
Bio6	Minimum temperature of the coldest month	7.2
S_oc	Subsoil organic carbon content	3.1
Slop	Slop	2.1
Bio7	Temperature annual range	1.3
S_clay	Subsoil clay content	0.9
Bio5	Maximum temperature of the warmest month	0.2
Bio2	Mean diurnal temperature range	0.2

**Figure 3:**
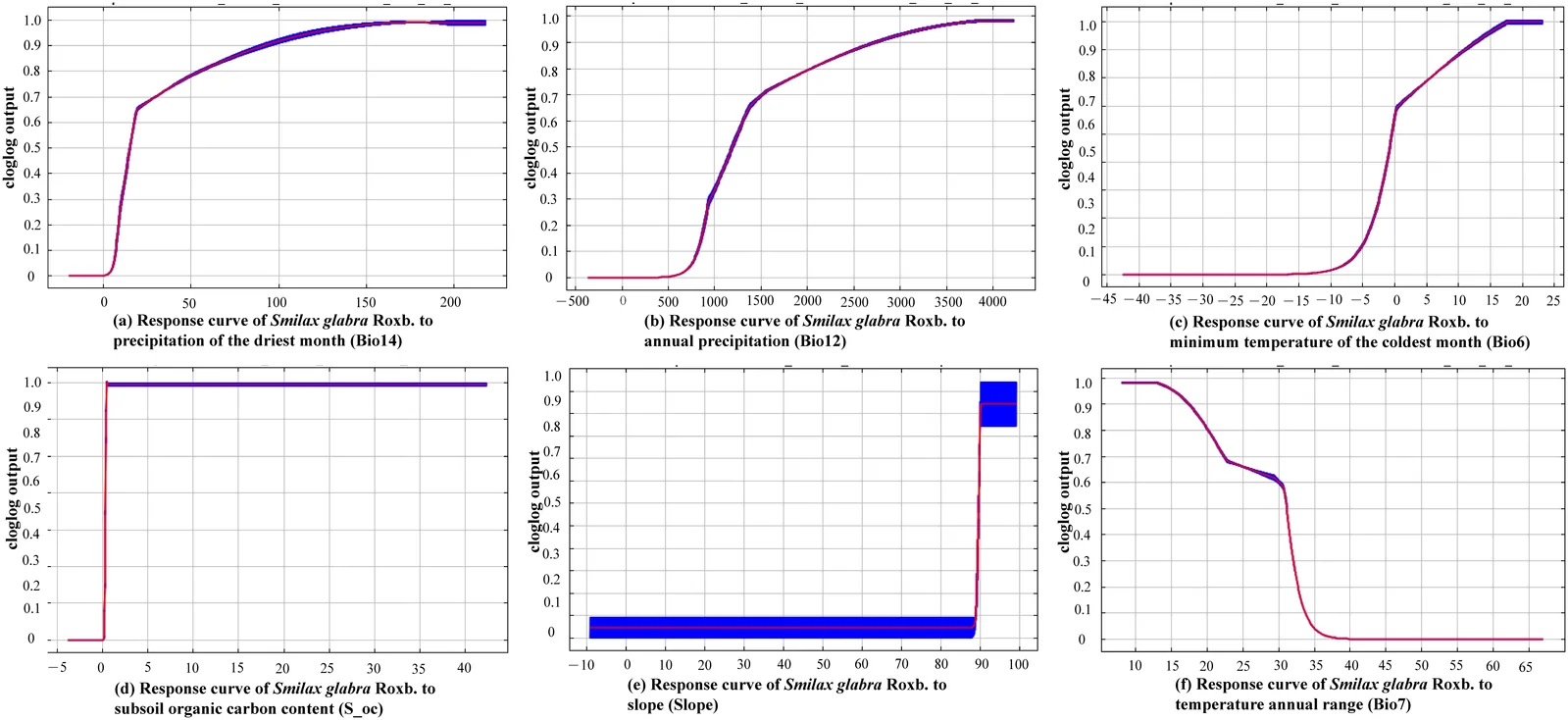
The response curve of dominant environmental factors: **(A)** Bio14 Precipitation of the driest month; **(B)** Bio12 Annual precipitation; **(C)** Bio6 Minimum temperature of the coldest month; **(D)** S_oc Subsoil organic carbon content; **(E)** Slop slop; **(F)** Bio7 Temperature annual range.

**Table 4:** The suitable range for the dominant environmental factors.

Variable	Suitable range	Adaptive threshold
Bio14	15.36 ~ 217.80 mm	178.35 mm
Bio12	1194.92 ~ 4222.90 mm	3846.12 ~ 4222.90 mm
Bio6	–0.34 ~ 23.11 °C	17.72 ~ 23.11 °C
S_oc	0.39 ~ 42.30	0.49
Slop	89.81 ~ 98.99%	90.14 ~ 98.99%
Bio7	8.06 ~ 31.06 °C	8.06 ~ 12.89 °C

### 3.3 Potential distribution pattern under current climatic conditions

The ensemble model projected the potential distribution of *S. glabra* under the current climate (**Figure 4**), which exhibited pronounced latitudinal zonation, being largely confined to the region south of the Yangtze River and reflecting a preference for warm, humid subtropical and tropical climates. Model evaluation metrics were robust, with AUC = 0.93, TSS = 0.77, and Kappa = 0.80.

**Figure 4:**
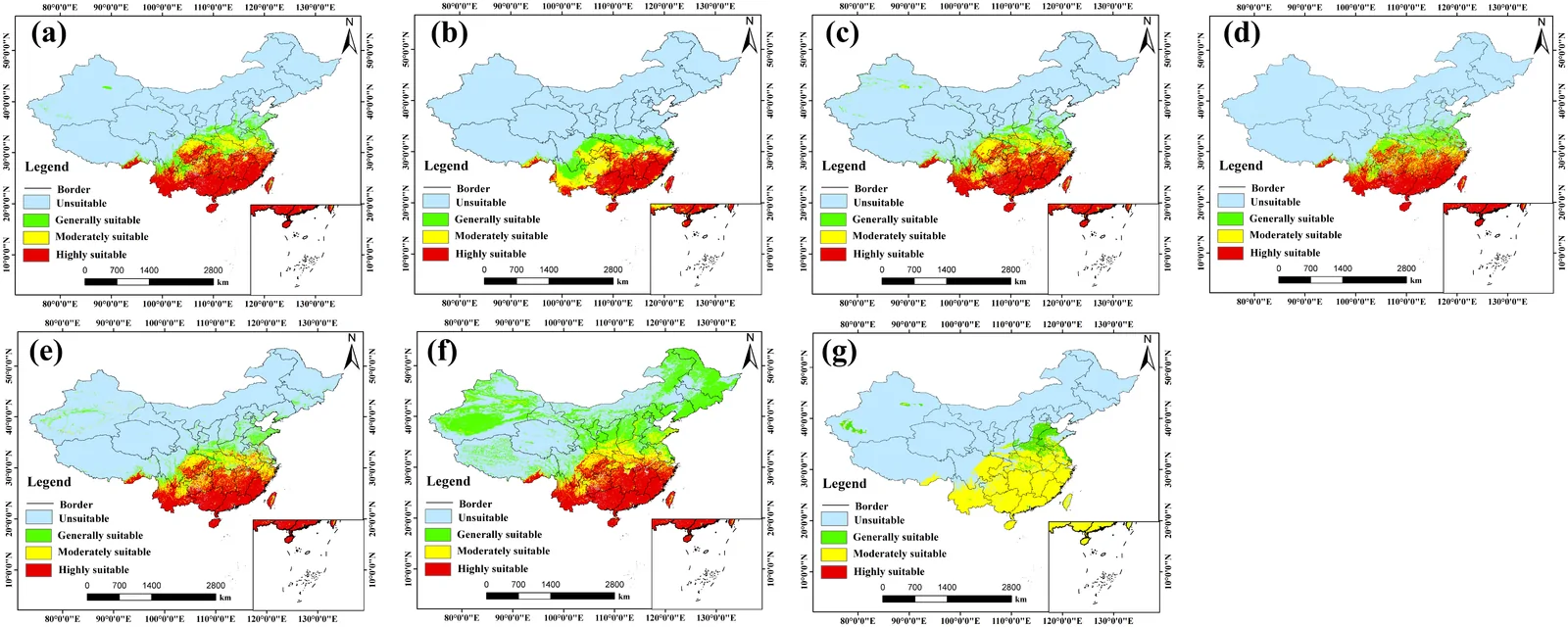
Potential distribution of *Smilax glabra* under current climatic conditions predicted by different modeling approaches. **(A)** Ensemble model; **(B)** MaxEnt; **(C)** GBM; **(D)** FDA; **(E)** GAM; **(F)** MARS; **(G)** CTA.

In terms of spatial pattern, the highly and moderately suitable areas constituted the core ecological niche of *S. glabra*, being extensively and predominantly distributed across South China (Guangdong, Guangxi, Hainan, Fujian, and Taiwan), Southwest China (Yunnan, Guizhou, Chongqing, southeastern Sichuan, and southeastern Tibet), and southern parts of Central and East China (e.g., Hunan, Jiangxi, Zhejiang, and southern Anhui). Generally suitable areas extended further north, crossing the middle and lower Yangtze Plain and reaching as far north as the Qinling-Huaihe line (covering southern Shaanxi, Henan, Shandong, and northern Jiangsu and Anhui). The total suitable area accounted for approximately 25.01% of China’s land area, whereas highly suitable areas represented only 13.90%, indicating a strong selectivity of this species for high-quality microhabitats.

Regarding single-model comparisons, the prediction patterns of the independently optimized MaxEnt model and the other algorithms retained in the ensemble were highly consistent with the ensemble results (**Supplementary Table 2**). Notably, during single-model evaluation, the excluded MARS model (**Figure 4**) showed obvious overgeneralization, anomalously extending its suitable area into arid and cold regions such as Xinjiang and Inner Mongolia. Quantitatively, the MARS model severely overestimated the total suitable area during the current period at 537.05 × 10^4^ km^2^, far exceeding the average of approximately 233.28 × 10^4^ km^2^, for the remaining algorithms. This likely stemmed from overfitting to extreme climatic thresholds. Consequently, the ensemble model constructed in this study effectively smoothed the spatial extrapolation bias of individual models. By relying on a weighting mechanism based on underlying evaluation metrics (AUC/TSS), it automatically penalized and excluded anomalous results such as those from MARS, thereby avoiding statistical misguidance that could arise from calculating an unweighted standard deviation (SD) or a simple arithmetic mean. Ultimately, the ensemble model provided a more robust, conservative, and ecologically plausible potential distribution pattern, offering a reliable baseline for subsequent assessments of climate change responses and Integrated quality regions.

### 3.4 Projected potential distribution under future climate scenarios

After applying a fixed-threshold strategy based on Max SSS, the main text focused on the most robust ensemble model (**Figure 5**) and the optimized MaxEnt model (**Figure 6**). Future projections from the remaining individual algorithms (GBM, FDA, GAM, MARS, and CTA) were placed in the Supplementary Materials as parallel references (**Supplementary Figures 5–9**). Cross-comparison revealed distinct spatiotemporal patterns for *S. glabra* under different SSPs. By incorporating algorithm-specific weights, the ensemble model smoothed extreme predictions from individual models and provided a more rigorous basis for trend assessment.

**Figure 5:**
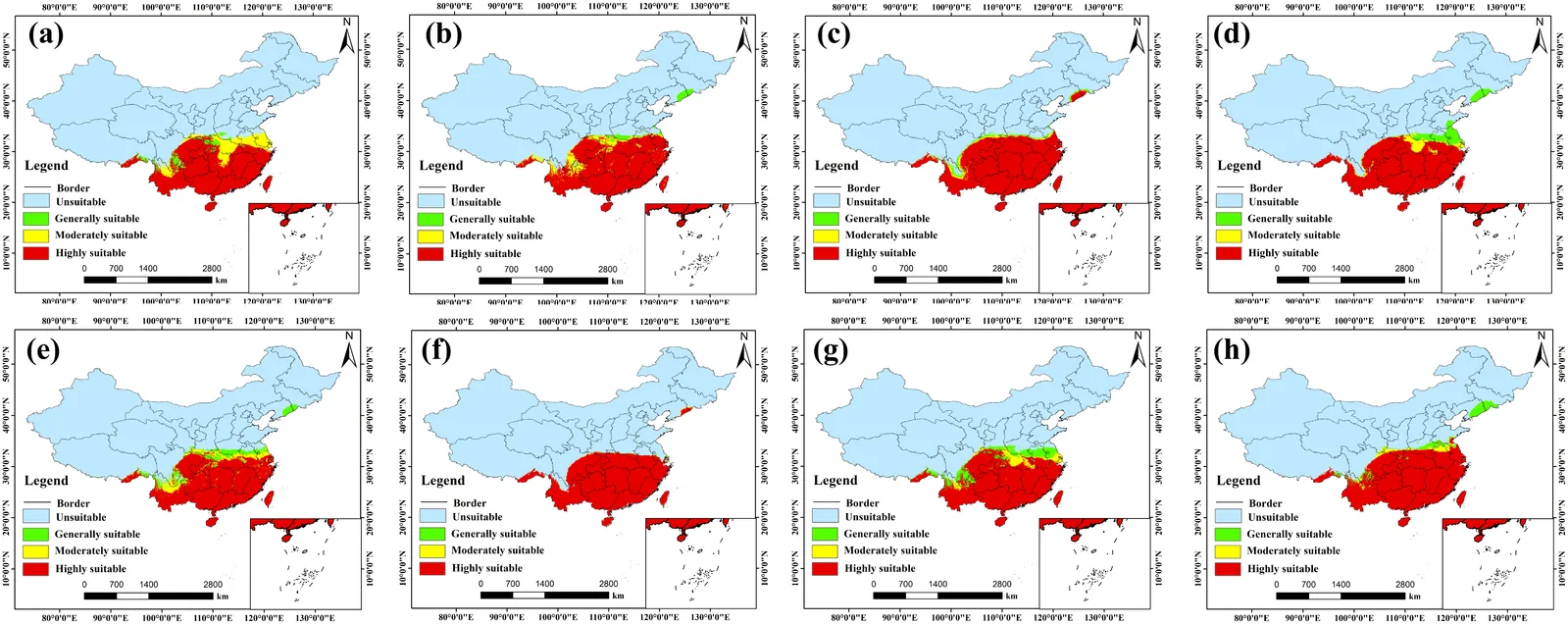
Potential distribution of *Smilax glabra* under future climate conditions as modelled by Ensemble model: **(A)** 2041~2060, SSP126; **(B)**2081~2100, SSP126; **(C)** 2041~2060, SSP245; **(D)** 2081~2100, SSP245; **(E)** 2041~2060, SSP370; **(F)** 2081~2100, SSP370; **(G)** 2041~2060, SSP585; **(H)** 2081~2100, SSP585.

**Figure 6:**
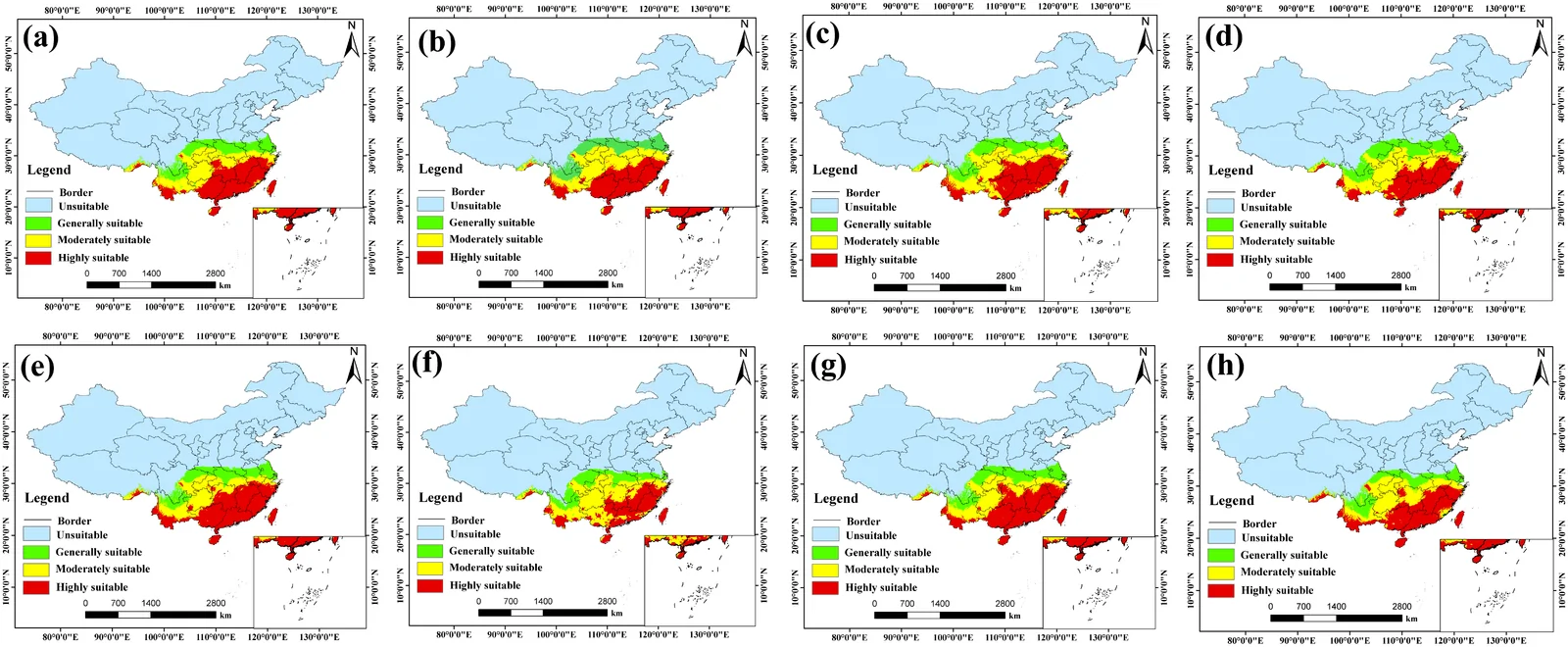
Potential distribution of *Smilax glabra* under future climate conditions as modelled by MaxEnt: **(A)** 2041~2060, SSP126; **(B)**2081~2100, SSP126; **(C)** 2041~2060, SSP245; **(D)** 2081~2100, SSP245; **(E)** 2041~2060, SSP370; **(F)** 2081~2100, SSP370; **(G)** 2041~2060, SSP585; **(H)** 2081~2100, SSP585.

Under the low-emission scenario (SSP126), *S. glabra* displayed considerable adaptive resilience. The ensemble model showed a slight contraction in total suitable area during the 2050s, followed by stabilization through the 2090s, whereas MaxEnt projected a modest expansion from the 2050s to the 2090s, with highly suitable habitat largely maintained (**Figure 7**). Fixed-threshold sensitivity analysis (**Supplementary Figure 10**) further supported the relative stability of suitable habitat under low emissions. For the high-emission scenario (SSP585), both the ensemble and MaxEnt models projected a marked expansion, rather than contraction, of highly suitable habitat for *S. glabra* by the 2090s. Area statistics showed that both highly suitable and total suitable areas increased appreciably relative to current conditions (**Supplementary Table 2**). These patterns imply that intense greenhouse gas forcing may broaden, rather than constrain, the climatic niche of *S. glabra*. More extensive areas with optimal temperature–moisture conditions and potential refugia to the southwest may largely explain this expansion.

**Figure 7:**
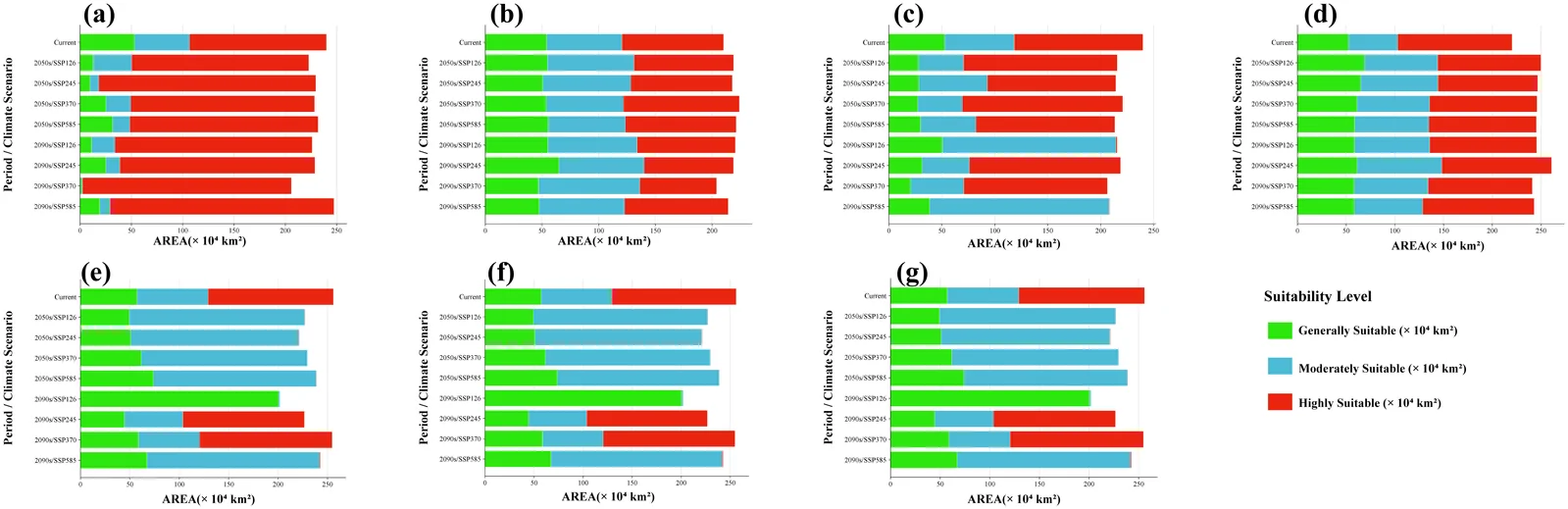
Suitable area of *Smilax glabra* under different future climate scenarios and periods: **(A)** Ensemble model; **(B)** MaxEnt; **(C)** GBM; **(D)** FDA; **(E)** GAM; **(F)** MARS; **(G)** CTA.

Spatially, delta maps of suitability probability (**Supplementary Figure 11**) further revealed heterogeneity in habitat change: positive values indicate habitat improvement, whereas negative values indicate degradation. Under SSP585 in the 2090s, localized negative patches remained in traditional core areas of South China (Guangdong, Guangxi, Fujian, and Hainan) and Southwest China (Yunnan, Guizhou, Chongqing, and southeastern Sichuan), reflecting local habitat degradation. Nevertheless, positive values predominated overall, especially toward the southwest and in areas with improved temperature–moisture conditions; the net effect was a marked expansion of highly suitable habitat. By integrating fixed classification thresholds with continuous probability comparisons, the ensemble model revealed the scenario-dependent response of *S. glabra* to future climate change: the species retains some adaptive potential under low emissions (SSP126), whereas under high emissions (SSP585) its climatically suitable range does not contract but instead expands. This dual sensitivity assessment offers more reliable consensus-based spatial evidence than single extreme projections.

### 3.5 Spatiotemporal dynamics of centroid shift in suitable habitats

Simulations of the suitable habitat centroid for *S. glabra* under future climate by individual and ensemble models (**Figure 8**; **Supplementary Table 3**) all indicated a pronounced southwestward migration trend. MaxEnt, GBM, FDA, and CTA consistently showed the centroid retreating southwestward from the junction of Hunan, Hubei, and Chongqing and surrounding areas toward the Yunnan–Guizhou Plateau and the margins of the Sichuan Basin, yet some algorithms produced extreme spatial jumps. For instance, FDA projected a migration distance of 600.45 km under SSP585 in the 2050s, while CTA projected up to 899.57 km under SSP245 in the 2050s, greatly exceeding the actual natural dispersal capacity of perennial plants.

**Figure 8:**
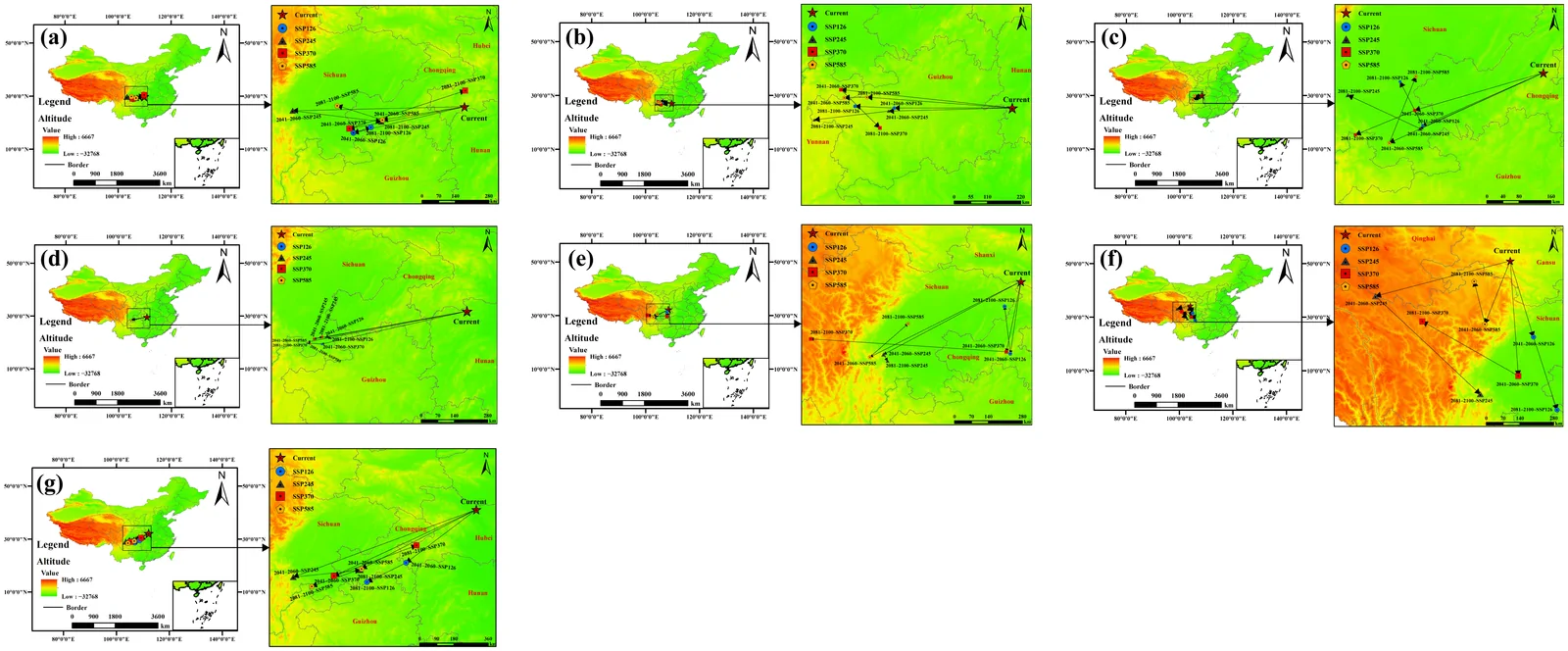
Centroid migration trajectories of the total suitable area for *Smilax glabra* under future climate scenarios: **(A)** Ensemble model; **(B)** MaxEnt; **(C)** GBM; **(D)** FDA; **(E)** GAM; **(F)** MARS; **(G)** CTA.

In contrast, the ensemble model effectively smoothed the algorithmic noise of individual models and provided more robust and ecologically plausible predictions (**Figure 8**). The ensemble centroid is currently located at approximately 109.80°E, 29.85°N and also migrates systematically southwestward in the future: in the 2050s, the migration distance relative to the current centroid was buffered and converged to between 304.42 km (SSP585) and 625.75 km (SSP245), indicating a marked but reasonable early climate response; by the 2090s, the centroid continued stepwise displacement from its 2050s position, with displacement magnitudes ranging from 68.64 km (SSP126) to 450.77 km (SSP370). This stepwise migration trajectory suggests that *S. glabra* habitats will undergo substantial spatial reorganization in the mid-21st century, followed by more complex local adjustments by the end of the century depending on emission pathways. The centroid migration direction reflected by the ensemble model aligns with most individual models, but it effectively filters out systematic bias. The robust predictions further support southwestward migration as the primary spatial strategy of *S. glabra* in response to climate change. The strategic importance of southwestern China as a core area for future germplasm conservation is thus highlighted.

### 3.6 Delineation of integrated quality regions

FDR-corrected Spearman correlation analysis (**Supplementary Figure 12**) showed that elevation (elev), topsoil silt content (t_silt), and topsoil pH (t_ph_h2o) were significantly positively correlated with astilbin content, whereas maximum temperature of the warmest month (Bio5), mean temperature of the warmest quarter (Bio10), and subsoil clay content (S_clay) were significantly negatively correlated.

Universal co-kriging interpolation based on Bio5, Bio10, and S_clay successfully characterized the spatial heterogeneity of astilbin content. LOOCV results (ME = -1.57, RMSE = 12.74, MSE = -0.116, RMSSE = 0.504; **Supplementary Table 4**) confirmed the statistical reliability of this spatial prediction model. In addition, combined with the kernel density map of sampling points (**Supplementary Figure 2**), areas at high risk of spatial extrapolation due to missing empirical data, such as central Jiangxi, were effectively identified. After excluding these uncertain areas, reliable high-value zones were mainly concentrated in southwestern Guizhou (**Figure 9A**). This pattern corroborates the regulatory effects of soil texture and thermal conditions on quality formation.

**Figure 9:**
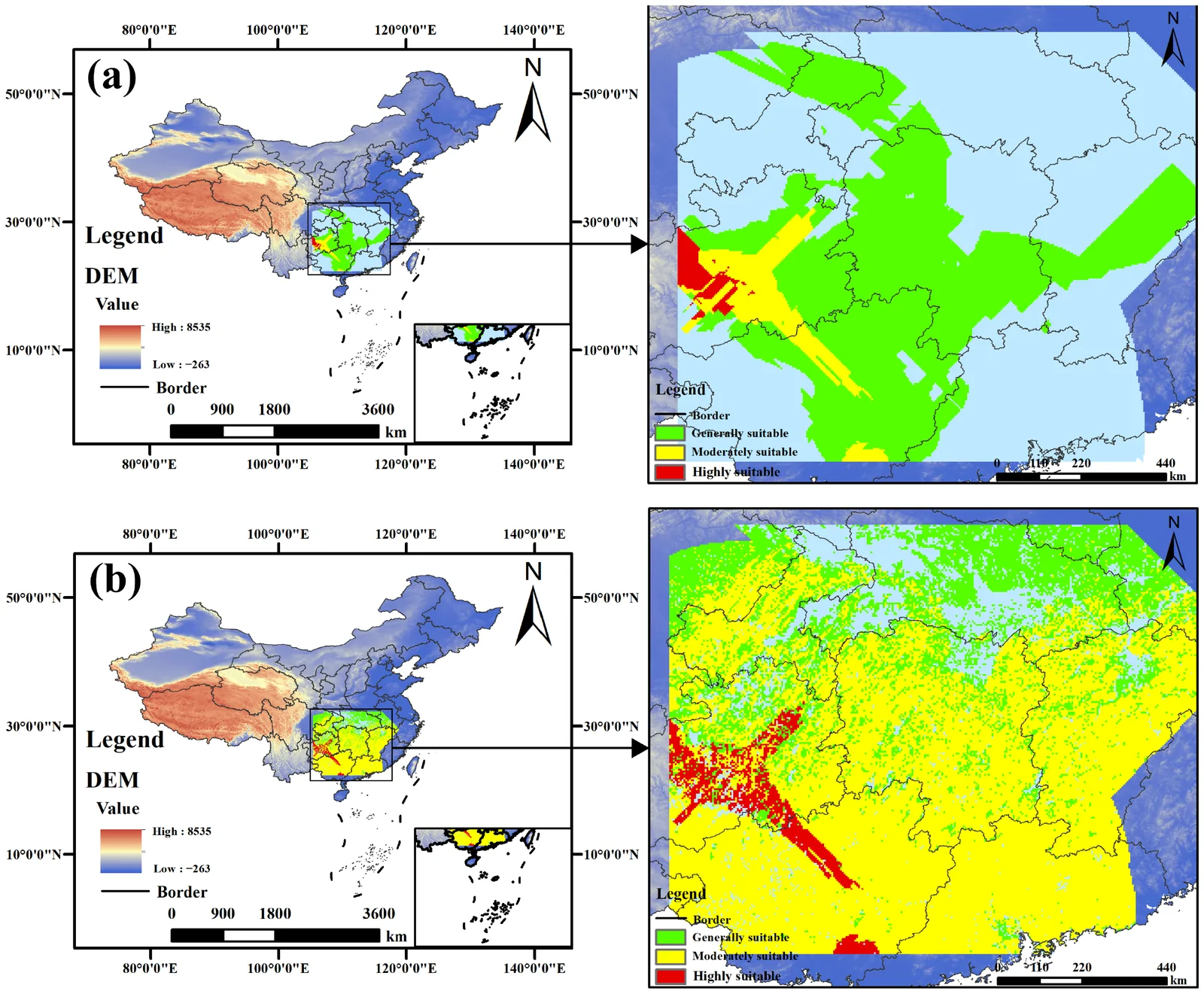
Integrated quality regions evaluation. **(A)** Co-kriging analysis of astilbin; **(B)** Integrated quality regions of *Smilax glabra*.

By integrating the chemical quality suitability index and the ecological suitability index through equal-weight overlay (0.5 each), an integrated quality assessment model that avoids spatial extrapolation risk was constructed (**Figure 9B**). To test the robustness of this equal-weight baseline assumption, a sensitivity analysis of weight allocation was further conducted by setting the ecological and chemical weights to 0.3:0.7 and 0.7:0.3, respectively (**Supplementary Figure 13**). The zoning results objectively showed that, regardless of weight shifts, southwestern Guizhou, northern Guangxi, and southeastern Guangxi consistently remained the optimal Integrated quality regions. This high spatial stability indicates that the assessment system is strongly robust to subjective weight assignment and genuinely achieves spatial synergy between suitable ecological niches and high metabolite accumulation, constituting core areas for the conservation and sustainable utilization of high-quality resources. Although the correlation analysis based on the current sample size is not yet sufficient to directly infer the underlying physiological regulatory mechanisms of quality formation, the model establishes a more rigorous and scientific production layout paradigm under the dual constraints of ecology and quality, providing clear spatial targets for subsequent controlled experiments to verify the true causal effects of these environmental factors.

## 4 Discussion

### 4.1 Methodological innovations and contributions of the integrated ecology–quality zoning framework

This work examined the habitat suitability, climate-change responses, and spatial variation in secondary metabolites of *Smilax glabra* Roxb. Although a two-dimensional Ecology-quality zoning framework that combines SDMs with spatial mapping of chemical constituents has been applied to other medicinal plants such as *Cyclocarya paliurus* and *Alpinia officinarum* (Jiang et al., 2025, 2026), two methodological gaps remain. First, chemical interpolation often lacks uncertainty constraints, which encourages unjustified spatial extrapolation into areas with few empirical samples. Second, assessments of future climate impacts frequently rely on dynamic classification thresholds (e.g., natural breaks), in which class boundaries shift with each scenario and may obscure the true direction of area change.

To address these issues, two improvements were made in the zoning procedure. For quality zoning, a joint assessment using KDE and kriging PSE was introduced to identify and exclude areas at high risk of spatial extrapolation, thereby placing the delineation of core high-quality areas on a stronger empirical footing. For future climate response, the Max SSS derived from the current ensemble model was adopted as a fixed absolute threshold, avoiding the bias associated with scenario-dependent dynamic classification. Continuous probability comparisons based on this fixed threshold (delta maps) showed that the direction of area change for *S. glabra* under future climate scenarios differed from that obtained with dynamic classification. Area statistics indicated that under the extreme high-emission scenario (SSP585), both highly suitable and total suitable areas by the 2090s increased relative to current conditions rather than contracting (**Supplementary Table 2**). These results suggest that intense greenhouse gas emissions may broaden rather than restrict the climatic niche of *S. glabra*, with more extensive optimal thermal–moisture conditions and potential refugia to the southwest likely contributing to this expansion. Thus, in addition to providing the first precise delineation of Integrated quality regions for *S. glabra*, this work improves the statistical rigor and ecological interpretability of the two-dimensional zoning framework for medicinal plants by incorporating uncertainty control and a fixed ecological threshold.

### 4.2 Dominant environmental factors and their ecophysiological mechanisms

Through environmental variable screening, correlation analysis, variance inflation factor testing, and the jackknife method to assess variable contributions, we identified precipitation of the driest month (Bio14), annual precipitation (Bio12), minimum temperature of the coldest month (Bio6), and subsoil organic carbon content (S_oc) as the core factors constraining the geographic distribution of *S. glabra*. Together, these four variables accounted for 95.2% of the total contribution. This outcome aligns with the species’ ecological nature as a subtropical, warmth- and moisture-loving medicinal plant that is highly sensitive to seasonal drought and low-temperature stress. Bio14 contributed the most (56%), making it the primary limiting factor, which matches the well-known dependence of medicinal plants with similar ecological habits on water availability (Li et al., 2025b). From an ecophysiological standpoint, precipitation availability during the driest month (typically winter to early spring in southern China) directly determines the germination of dormant buds on the underground rhizomes of *S. glabra* and the early vegetative growth of newly emerged vines. If extreme drought occurs during this period, shallow rhizomes undergo severe dehydration, and carbohydrate remobilization and translocation from rhizomes to new aboveground tissues are impeded, ultimately constraining population expansion and establishment. With regard to temperature stress, the lower suitable limit of the minimum temperature of the coldest month is −0.34 °C. This near-freezing tolerance threshold points to a relatively weak cold acclimation capacity in *S. glabra*. As a typical subtropical liana, it lacks deep frost-resistance mechanisms; when winter temperatures drop below freezing, its cell membrane system is prone to severe lipid peroxidation, causing massive electrolyte leakage and necrosis of overwintering dormant buds. This physiological barrier fundamentally restricts the natural expansion of *S. glabra* into higher-latitude and frigid regions (Sun et al., 2026). Among soil factors, subsoil organic carbon content (S_oc), as the fourth dominant factor, not only enhances water and nutrient retention by improving soil aggregate structure, but also directly intervenes in secondary metabolite accumulation via complex rhizosphere microecology–plant signaling interactions. Abundant subsoil organic carbon supplies ample carbon sources for rhizosphere and endophytic microorganisms. The activity of microbial communities can specifically upregulate the expression of key enzyme genes (e.g., PAL and CHS) in the phenylpropanoid and flavonoid biosynthetic pathways in *S. glabra* by triggering systemic acquired resistance (SAR) or acting as exogenous elicitors, thereby markedly promoting the targeted enrichment of bioactive flavonoids such as astilbin in rhizomes (Jia et al., 2024). Notably, soil organic carbon—a key indicator of soil quality and ecosystem function—is modulated by climate, vegetation, and human management (Shu et al., 2024). In this study, its role reflects local habitat regulation, further highlighting that the distribution pattern of *S. glabra* is primarily shaped by macroclimate and secondarily coordinated by micro-scale edaphic factors.

### 4.3 Advantages of the ensemble framework and methodological considerations

In species distribution modeling, a single algorithm is susceptible to its intrinsic assumptions and data sensitivity, which can cause predictions to deviate from a species’ true niche (Peterson et al., 2018). Multiple studies have shown that ensemble models significantly outperform single models across various species, offering higher predictive stability and ecological interpretability (Mahmoud et al., 2025; Li et al., 2026a). For *S. glabra*, a traditional medicinal plant whose geographic distribution is governed largely by temperature and precipitation, an inadequately calibrated single model may generate unreasonable inferences in marginal habitats.

The empirical data in this study substantiate the core advantage of the ensemble framework in correcting single-algorithm bias. In estimating suitable areas and predicting spatial patterns under the current climate, the single MARS model, owing to overfitting to extreme climatic thresholds, produced an anomalous total area as high as 537.05 × 10^4^ km^2^, unreasonably extrapolating suitable habitat into arid and frigid regions such as Xinjiang and Inner Mongolia. By contrast, the ensemble model, through AUC- and TSS-based weighting, strictly removed such spatial artifacts and robustly converged the total suitable area to 240.07 × 10^4^ km^2^, tightly and accurately confined to the true niche south of the Yangtze River. Moreover, in predicting centroid migration trajectories, the empirical data also exposed the instability of single models (**Supplementary Table 3**). Some single models exhibited extreme spatial jumps under future scenarios; for example, the CTA model showed a centroid shift as large as 899.57 km under SSP245 in the 2050s and 847.34 km under SSP585 in the 2090s, greatly exceeding the natural dispersal limits of perennial plants. The ensemble model effectively smoothed such algorithmic noise, yielding more conservative and biologically plausible migration distances (buffered to 625.75 km and 175.57 km under the two scenarios, respectively). These quantitative comparisons based on predicted area, centroid displacement, and spatial pattern clearly indicate that the ensemble framework, through weighted integration of multiple algorithms, effectively suppresses systematic bias and markedly improves ecological realism (Koç, 2025). Furthermore, parameter optimization of MaxEnt using the ENMeval package reduced environmental variables from 63 to 22 key factors, significantly narrowing the fit difference between training and validation sets and mitigating overfitting risk (Bai et al., 2024). This optimization strategy maintained robust predictive capability under small sample size and mixed wild–cultivated data, confirming the key role of parameter tuning in improving the generalization performance of niche models (Jiang et al., 2026b).

In addition, in Biomod2 ensemble modeling, the number of pseudo-absence points strongly affects class balance in machine learning algorithms. Although tens of thousands of background points are often used in a single MaxEnt model, for ensemble algorithms relying on presence–absence data (e.g., Random Forest and Artificial Neural Networks), an extreme positive-to-negative ratio (e.g., 5,000 or 10,000 pseudo-absence points) causes severe class imbalance, biasing predictions toward the majority class (“absence”) and severely underestimating suitable habitat. Based on 571 effective distribution records, this study set 1,000 pseudo-absence points. A ratio of approximately 1:2 ensures balanced representation of environmental data when distinguishing suitable from unsuitable habitat, thereby reducing spatial bias while avoiding statistical penalties caused by pseudo-absences dominating the model. Combined with multiple rounds of cross-validation and repeated runs, this strategy maintains algorithmic robustness while ensuring predictive reliability, in line with the methodological consensus of recent studies using moderately balanced pseudo-absences to optimize ensemble model performance.

Although the ensemble model constructed in this study shows high macro-scale predictive accuracy, the inherent data and methodological limitations of SDMs warrant objective scrutiny. First, because some distribution data were derived from public databases, the samples inevitably mix wild and cultivated individuals. Therefore, the suitable areas predicted here tend to reflect the fundamental niche of *S. glabra* and its potential cultivable suitability, rather than its future purely natural, climate-driven wild population migration trajectory, which should be interpreted with due caution. Second, regarding sampling bias correction, although strict spatial rarefaction was performed on distribution records before modeling to minimize collection bias and spatial autocorrelation, residual bias may remain compared with introducing a bias surface or applying target-group background restriction. In addition, traditional random splitting was used for cross-validation. As recent studies have noted, when residual spatial autocorrelation exists in species distributions, random splitting may overestimate predictive performance to some extent. Future SDMs studies should prioritize spatial block cross-validation combined with stricter discrimination between wild and cultivated data, so as to further enhance ecological realism and absolute robustness of predictions.

### 4.4 Spatiotemporal dynamics and vulnerability of suitable habitats under climate change scenarios

Simulations under different future climate scenarios indicate that greenhouse gas emission intensity markedly alters the area, configuration, and persistence of suitable habitat for *S. glabra*. By adopting the Max SSS under current climate as a fixed absolute biological benchmark, together with continuous suitability change maps (Delta Maps), this study reveals the scenario-dependent response of the species to future climate change. Under the low-emission scenario (SSP126), although global warming is relatively moderate (Feng et al., 2026), the suitability probability in its historical core distribution still shows a widespread absolute decline. Under intermediate emission scenarios (SSP245 and SSP370), suitable habitat contracts markedly and fragmentation intensifies. Under the extreme high-emission scenario (SSP585), persistent warming and disrupted precipitation patterns produce complex redistribution of water and heat (Gillani et al., 2025). Area statistics based on the fixed absolute threshold show that by the 2090s, both highly suitable and total suitable areas for *S. glabra* increase relative to current conditions rather than contract (**Supplementary Table 2**). This suggests that intense greenhouse gas emissions may broaden rather than constrict the climatic niche of *S. glabra*, and that more extensive optimal hydrothermal conditions and potential refugia to the southwest may be the main drivers of this expansion. According to the “large genome constraint hypothesis,” species with large genomes are restricted in extreme environments (Knight et al., 2005). This hypothesis has been tested in Liliaceae and Zingiberaceae, where large-genome species tend to occur in humid, warm environments with longer growing seasons and are excluded from arid, strongly seasonal climates (Carta and Peruzzi, 2016; Záveská et al., 2024). These patterns suggest that combined heat and drought stress may limit the distribution and survival space of large-genome groups, such as perennial herbs and lianescent medicinal plants; however, the expansion of *S. glabra* under SSP585 in this study indicates that this constraint may be locally offset when hydrothermal conditions improve in some regions. In terms of spatial shifts, the centroid of suitable habitat shows a broad southwestward trend. At present, this centroid lies at the junction of Hunan, Hubei, and Chongqing and adjacent areas; under different climate scenarios, it gradually shifts toward high-elevation inland areas in Guizhou, Guangxi, and Yunnan. It should be emphasized that because future climate projections in this study are based on a single global climate model (GCM), BCC-CSM2-MR, the reported migration distances (e.g., shifts of several hundred kilometers) should be regarded as estimates under this specific climate forcing rather than physically precise distances. Although BCC-CSM2-MR performs reasonably well in simulating the East Asian monsoon and precipitation patterns, the direction of centroid shifts still differs markedly among plant groups: suitable habitat for some karst plants contracts toward higher elevations and latitudes (Yao et al., 2024), whereas *S. glabra* shows a relatively consistent southwestward tendency. The mountainous areas of Southwest China, with their favorable hydrothermal configuration, may serve as potential refugia for moisture-loving plants under climate warming (Guo et al., 2025b). Unlike some medicinal plants in Liliaceae that shift northwestward in alpine regions (Dong et al., 2025), moisture-loving species such as *S. glabra* tend to disperse toward lower-latitude, moderately elevated mountainous areas in Southwest China with relatively balanced hydrothermal conditions; this broad trend is consistent with the refugial role of these mountains. Notably, *S. glabra* reproduces mainly asexually via rhizomes and has weak natural dispersal ability. Moreover, wild populations have long been disturbed by anthropogenic harvesting and increasing habitat fragmentation. Its actual migration rate is therefore likely to lag behind the shift of climatically suitable areas. Newly suitable areas in Southwest China may thus be unlikely to form stable natural populations, making assisted *ex situ* conservation and germplasm introduction urgently needed.

### 4.5 Limitations of the technical system and future improvement directions

The technical framework developed in this study—integrating multi-model ensemble ecological simulation, co-kriging quality interpolation, and weighted overlay integrated zoning—refines the research paradigm for Integrated quality regions of medicinal and edible plants and offers a methodological reference for studies on similar species. Nevertheless, several limitations remain. First, only 30 astilbin content samples were available, and their spatial distribution was uneven. Although kernel density analysis and prediction standard error mapping were used to rigorously delineate areas at risk of spatial extrapolation, thereby ensuring the robustness of the conclusions, the existing data remain insufficient to characterize local, fine-scale quality variation. Future work could expand the sampling range and incorporate biological factors such as community microhabitat, endophytic fungi, and rhizosphere microorganisms to dissect the multi-level regulatory network linking environment, microorganisms, and quality. Second, this study did not distinguish quality differences between wild and cultivated populations, nor did it consider the effects of land use type, pests and diseases, or anthropogenic disturbance on suitable habitats and quality. Finally, future research could couple multiple global climate models to compare prediction differences and reduce simulation uncertainty arising from a single climate model, while incorporating species dispersal models to quantify the limiting distance of natural migration, thereby providing finer support for germplasm conservation strategies under climate change.

### 4.6 Conservation and industrial utilization strategies based on zoning results

Based on the findings of this study and the current state of the *S. glabra* industry, the following conservation and utilization recommendations are proposed. First, southwestern Guizhou and northern and southeastern Guangxi should be designated as core high-quality germplasm conservation areas, where *in situ* conservation is implemented, uncontrolled harvesting is strictly prohibited, and wild high-quality populations are protected. For “spatial extrapolation areas” such as central Jiangxi and southern Hunan, where model-predicted suitability is high but empirical data are lacking, large-scale field surveys of germplasm resources and sampling validation should be prioritized to clarify their true development potential. Second, although the climatically suitable area for *S. glabra* generally expands under the extreme high-emission scenario (SSP585), given its weak natural dispersal ability and the likelihood that its actual migration rate lags behind the shift of suitable areas, attention should still focus on potential newly suitable areas and refugia in the eastern Yunnan–Guizhou Plateau and other parts of Southwest China. *Ex situ* germplasm conservation and introduction and domestication should be initiated in advance, and backup cultivation bases should be established. Third, although traditional production areas (Guangdong and southern Fujian) have relatively high ecological suitability, their astilbin accumulation capacity is moderate. Cultivation management should be optimized and soil texture and pH improved to enhance medicinal quality in a targeted manner. Fourth, although climatically suitable areas may expand under high-emission scenarios, warming still entails uncertainties such as disrupted hydrothermal patterns and localized habitat degradation. From the perspective of long-term industry development, low-carbon development models should still be promoted to slow regional climate change and protect the living environment of *S. glabra* at its source. In summary, this study completed an integrated quality zoning of *S. glabra* through a multi-model assessment framework, balancing ecological conservation, quality improvement, and industrial development. It provides scientific support for the sustainable utilization of this important medicinal and edible resource.

## 5 Conclusions

A multi-model ensemble framework with a rigorously fixed threshold (incorporating the optimized MaxEnt algorithm) was combined with kernel density estimation for uncertainty assessment and kriging interpolation. This approach allowed us to systematically characterize the habitat constraints of *S. glabra*, the spatiotemporal dynamics of its suitable areas under future climate scenarios, and the spatial distribution of quality indicators, and to delineate Integrated quality regions for the species. Precipitation and temperature emerged as key climatic determinants of its distribution. The species showed weak tolerance to both cold and drought, with natural populations largely confined to the subtropical humid zone south of the Yangtze River. Under future climate scenarios, sensitivity analyses based on a fixed biological baseline and continuous probability comparisons revealed that *S. glabra* retains some adaptive potential under the low-emission scenario (SSP126). In contrast, under the high-emission scenario (SSP585), area estimates indicated that both highly suitable and total suitable areas increased relative to current conditions, suggesting a possible broadening rather than contraction of its climatic niche; spatially, the centroid of suitable habitat shifted southwestward toward Yunnan and Guizhou. By integrating the spatial distribution of astilbin content with ecological suitability assessment, and after rigorously excluding areas at high risk of spatial extrapolation, the analysis further identified southwestern Guizhou, northern Guangxi, and southeastern Guangxi as the optimal Integrated quality regions for *S. glabra*. These areas show close coupling between ecological conditions and medicinal quality formation and merit priority for developing and conserving high-quality resources. Constrained by limited sample sizes for chemical indicators and model assumptions, this work quantified uncertainty statistically as far as possible, yet further refinement is needed. Future sampling should focus on the gaps revealed here, such as parts of eastern and central China’s marginal production areas, to validate and improve the multi-model simulation framework. Overall, the highly quantitative, classification-bias-avoiding Ecology-quality zoning approach developed here can provide more robust and rigorous theoretical support and practical guidance for germplasm conservation and adaptive cultivation of *S. glabra* under climate change.

## Data Availability

The original contributions presented in the study are included in the article/**Supplementary Material**. Further inquiries can be directed to the corresponding authors.

## References

[B1] Allouche O. Tsoar A. Kadmon R. (2006). Assessing the accuracy of species distribution models: prevalence, kappa and the true skill statistic (TSS). J. Appl. Ecol. 43, 1223–1232. doi: 10.1111/j.1365-2664.2006.01214.x40046247

[B2] Bai J. Wang H. Hu Y. (2024). Prediction of potential suitable distribution of liriodendron chinense (Hemsl.) sarg. in China based on future climate change using the optimized maxEnt model. Forests 15, 988. doi: 10.3390/f1506098830654563

[B3] Buisson L. Thuiller W. Casajus N. Lek S. Grenouillet G. (2010). Uncertainty in ensemble forecasting of species distribution. Glob. Change Biol. 16, 1145–1157. doi: 10.1111/j.1365-2486.2009.02000.x40046247

[B4] Carta A. Peruzzi L. (2016). Testing the large genome constraint hypothesis: plant traits, habitat and climate seasonality in L iliaceae. New Phytol. 210, 709–716. doi: 10.1111/nph.13769 26595257

[B5] Dong P. Wang L. Wang L. Lei M. Qiu D. Bai G. . (2025). Investigating the distributional response of the rare and endangered plant Fritillaria przewalskii to climate change based on optimized MaxEnt model. Sci. Rep. 15, 35939. doi: 10.1038/s41598-024-75872-6 41093917 PMC12528763

[B6] Feng B. Nie S. Wu H. Wen Y. Wang H. Zhang G. . (2026). Increased groundwater recharge under climate change will enhance nitrogen fixation in groundwater-dependent ecosystems. Water Res. 299, 125873. doi: 10.1016/j.watres.2026.125873 41962233

[B7] Gillani S. W. Ahmad M. Manzoor M. Al-Andal A. Khan R. W. A. Ayoola J. O. . (2025). Integrating environmental variables and biotic interactions to predict the future distribution of the endangered Aconitum heterophyllum Wall. ex Royle. BMC Plant Biol. 26, 45. doi: 10.1186/s12870-025-07859-y 41339780 PMC12781508

[B8] Guo J. X. Wu Y. Zhang T. D. Lv F. L. Gong Y. F. (2025a). Dual niche modeling with GEE and SHAP for predicting habitat shifts of Haloxylon ammodendron and Cistanche deserticola under climate change. PloS One 20, e0338809. doi: 10.1371/journal.pone.0338809 41417741 PMC12716759

[B9] Guo M. Zeng J. Wang Z. Shen Y. (2024). Advances in the chemical constituents, pharmacological activity, and clinical application of Smilacis Glabrae Rhizoma: A review and predictive analysis of quality markers (Q-markers). Heliyon 10, e29557. doi: 10.1016/j.heliyon.2024.e29557 38644901 PMC11031832

[B10] Guo S. Y. Zhang Y. Chen X. H. Zhang Y. W. Cao B. B. Zhu S. C. . (2026). Protective role and mechanism of flavonoids from Smilax glabra Roxb. against lead-induced neurotoxicity and hematotoxicity in Daniorerio. J. Ethnopharmacol. 364, 121472. doi: 10.1016/j.jep.2026.121472 41791617

[B11] Guo X. Bai W. Wang Y. Hao S. Zhao L. Li X. . (2025b). Predicting habitat suitability for an endangered medicinal plant, Saussurea medusa: insights from ensemble species distribution models. Front. Plant Sci. 16, 1590206. doi: 10.3389/fpls.2025.1590206 40630732 PMC12236372

[B12] Harrigan E. (2025). Integrating GIS and Multi-Criteria Decision Analysis for Conservation Modeling in the Southern Appalachians (Durham, NC: Duke University).

[B13] Hua S. Zhang Y. Liu J. Dong L. Huang J. Lin D. . (2018). Ethnomedicine, phytochemistry and pharmacology of smilax glabra: an important traditional chinese medicine. Am. J. Chin. Med. 46, 261–297. doi: 10.1142/S0192415X18500143 29433390

[B14] Huang B. Chen S. Xu L. Jiang H. Chen X. He H. . (2024). Predicting the potential geographical distribution of Zingiber striolatum Diels (Zingiberaceae), a medicine food homology plant in China. Sci. Rep. 14, 22206. doi: 10.1038/s41598-024-73202-4 39333747 PMC11436980

[B15] Huang J. Ling C. X. (2005). Using AUC and accuracy in evaluating learning algorithms. IEEE Trans. Knowl. Data Eng. 17, 299–310. doi: 10.1109/TKDE.2005.5025079929

[B16] Jeeno P. Tongban S. Yana P. Wongta A. Sutan K. Yadoung S. . (2022). Tentative Identification of Phytochemicals from Smilax glabra and Smilax corbularia Extracts by LC-QTOF/MS and Their Bioactive Potential. Plants (Basel). 11, 2089. doi: 10.3390/plants11162089 36015393 PMC9415527

[B17] Jia N. Li L. Guo H. Xie M. (2024). Important role of Fe oxides in global soil carbon stabilization and stocks. Nat. Commun. 15, 10318. doi: 10.1038/s41467-024-54832-8 39609446 PMC11605054

[B18] Jiang H. Chen H. Wang H. Huang B. Chen T. (2025). Establishment of integrated quality regions for the rare medicine food homology plant cyclocarya paliurus (Batal.) iljinsk in china. Biol. (Basel). 14, 1639. doi: 10.3390/biology14121639 41463414 PMC12729511

[B19] Jiang H. Huang B. Li T. Liu Y. Zhang S. Yang Q. . (2026a). Zoning of integrated quality regions for alpinia officinarum hance based on a multi-model evaluation system. Biol. (Basel). 15, 369. doi: 10.3390/biology15040369 41744678 PMC12937820

[B20] Jiang H. Zhang Y. Li T. Zhang S. Liu Y. Chen Y. . (2026b). Prediction of potential habitat distribution of cibotium barometz (L.) J. Sm. Under climate change based on a multi-model ensemble framework. Biol. (Basel). 15, 692. doi: 10.3390/biology15090692 42117831 PMC13162567

[B21] Knight C. A. Molinari N. A. Petrov D. A. (2005). The large genome constraint hypothesis: evolution, ecology and phenotype. Ann. Bot. 95, 177–190. doi: 10.1093/aob/mci011 15596465 PMC4246716

[B22] Koç İ. (2025). Predicting the potential geographic distribution of Fagus orientalis Lipsky under climate change using an ensemble model approach in Türkiye. Naturwissenschaften 112, 93. doi: 10.1007/s00114-025-02051-6 41324682

[B23] Li J. Huang Y. Pan Y. Zhao C. Yang Y. Yang J. (2026a). Prediction of potential suitable habitats of cupressus duclouxiana under climate change based on biomod2 ensemble models. Biol. (Basel). 15, 165. doi: 10.3390/biology15020165 41594900 PMC12837535

[B24] Li K. Wang X. Liu Z. Xue C. (2025a). Characteristics and driving mechanisms of vegetation phenology variations in the Bosten Lake Basin, Xinjiang, China. PloS One 20, e0332896. doi: 10.1371/journal.pone.0332896 40997054 PMC12463244

[B25] Li P. Tedersoo L. Crowther T. W. Dumbrell A. J. Dini-Andreote F. Bahram M. . (2023). Fossil-fuel-dependent scenarios could lead to a significant decline of global plant-beneficial bacteria abundance in soils by 2100. Nat. Food 4, 996–1006. doi: 10.1038/s43016-023-00869-9 37904026

[B26] Li S. Long C. Li M. Tian L. Li R. Guo J. . (2026b). Identification of chemical constituents of Smilax glabra Roxb. and comparative study on pharmacokinetics of 12 main bioactive components in normal and hyperuricemia rats by UPLC-Q-Exactive Orbitrap-HRMS method. J. Pharm. Biomed. Anal. 267, 117149. doi: 10.1016/j.jpba.2025.117149 40972479

[B27] Li Y. Li Y. Zeng P. Zhang W. Jiang W. Yang Z. (2001). “ Current status of the Smilax glabra medicinal material market”, in: Proceedings of the Academic Conference on Modernization of Traditional Chinese Medicine (Beijing: China Association of Chinese Medicine), 111–113.

[B28] Li Y. Li Y. Zeng P. Zhang W. Jiang W. Yang Z. (2002). Investigation on plant resources of Smilax glabra. Chin. Tradit. Herb. Drugs 33, 850–852.

[B29] Li Y. Zhang Y. Wu Y. Chen Z. Liao M. Yu Y. . (2025b). The impact of soil moisture stress on the growth and biosynthesis of effective components in Salvia miltiorrhiza. Ind. Crops Prod. 225, 120529. doi: 10.1016/j.indcrop.2024.12052942574925

[B30] Liang W. Liu J. Mo Y. Cao Y. Qin F. Feng S. . (2025). Seedling propagation and cultivation techniques of Smilax glabra Roxb. China Trop. Agric., 83–88.

[B31] Liang X. Zhang T. (2026). Scientific research on intercropping cultivation and growth regulation technology of Smilax glabra under pine forest. Seed. World, 186–188.

[B32] Mahmoud A. R. Farahat E. A. Hassan L. M. Halmy M. W. A. (2025). Predicting the future impact of climate change on the distribution of species in Egypt's mediterranean ecosystems. BMC Plant Biol. 25, 644. doi: 10.1186/s12870-025-06630-7 40375130 PMC12079925

[B33] Muscarella R. Galante P. J. Soley‐Guardia M. Boria R. A. Kass J. M. Uriarte M. . (2014). ENM eval: An R package for conducting spatially independent evaluations and estimating optimal model complexity for Maxent ecological niche models. Methods Ecol. Evol. 5, 1198–1205. doi: 10.1111/2041-210X.1226140046247

[B34] Peterson A. T. Cobos M. E. Jiménez-García D. (2018). Major challenges for correlational ecological niche model projections to future climate conditions. Ann. N. Y. Acad. Sci. 1429, 66–77. doi: 10.1111/nyas.13873 29923606

[B35] Pradhan P. (2016). Strengthening MaxEnt modelling through screening of redundant explanatory bioclimatic variables with variance inflation factor analysis. Researcher 8, 29–34.

[B36] Regos A. Gagne L. Alcaraz-Segura D. Honrado J. P. Domínguez J. (2019). Effects of species traits and environmental predictors on performance and transferability of ecological niche models. Sci. Rep. 9, 4221. doi: 10.1038/s41598-019-40766-5 30862919 PMC6414724

[B37] Shi W. Wu Z. Wu J. Jia M. Yang C. Feng J. . (2023). A comprehensive quality control evaluation for standard decoction of Smilax glabra Roxb based on HPLC-MS-UV/CAD methods combined with chemometrics analysis and network pharmacology. Food Chem. 410, 135371. doi: 10.1016/j.foodchem.2022.135371 36608559

[B38] Shu X. Gao L. Yang J. Xia J. Song H. Zhu L. . (2024). Spatial distribution characteristics and influencing factors of soil organic carbon based on the geographically weighted regression model. Environ. Monit. Assess. 196, 1083. doi: 10.1007/s10661-024-13279-6 39432014

[B39] Sun X. Guan J. Meng L. Wang C. Chen N. Wei T. . (2026). Predicting future habitat suitability of Smilax glabra under climate change scenarios. Sci. Rep. 16, 16306. doi: 10.1038/s41598-026-36064-6 42020482 PMC13213053

[B40] Tavan M. Azizi A. Sarikhani H. Dastres E. Rigano M. M. Mirjalili M. H. (2025). Predicting the impact of climate change on the habitat suitability and phytochemical quality of Salvia multicaulis Vahl. BMC Plant Biol. 26, 61. doi: 10.1186/s12870-025-07756-4 41353141 PMC12798074

[B41] Thuiller W. (2003). BIOMOD–optimizing predictions of species distributions and projecting potential future shifts under global change. Glob. Change Biol. 9, 1353–1362. doi: 10.1046/j.1365-2486.2003.00666.x 25200636 PMC4340559

[B42] Tian C. Yu M. Fang Y. Zhao Y. Fu L. Chen J. . (2025). Therapeutic effect of smilax glabra roxb. on weaning rats against pb-induced nephrotoxicity based on network pharmacology. Biol. Trace Elem. Res. 203, 2714–2727. doi: 10.1007/s12011-024-04366-3 39240306

[B43] Wang S. Fang Y. Yu X. Guo L. Zhang X. Xia D. (2019). The flavonoid-rich fraction from rhizomes of Smilax glabra Roxb. ameliorates renal oxidative stress and inflammation in uric acid nephropathy rats through promoting uric acid excretion. Biomed. Pharmacother. 111, 162–168. doi: 10.1016/j.biopha.2018.12.050 30579255

[B44] Wei Z. Ni X. Cui H. Shu C. Peng Y. Liu J. . (2025). Engeletin attenuates the inflammatory response via inhibiting TLR4-NFκB signaling pathway in Crohn's disease-like colitis. J. Ethnopharmacol. 336, 118733. doi: 10.1016/j.jep.2024.118733 39181281

[B45] Wu Y. Liu X. Su H. He L. Yang Q. Li R. . (2026). Colloidal self-assembly of astilbin with smilax glabra polysaccharides enables carrier-free delivery and improved anti-inflammatory performance. Colloids. Surf. B. Biointerfaces. 259, 115286. doi: 10.1016/j.colsurfb.2025.115286 41273892

[B46] Wu H. Wang Y. Zhang B. Li Y. L. Ren Z. X. Huang J. J. . (2022). Smilax glabra roxb.: A review of its traditional usages, phytochemical constituents, pharmacological properties, and clinical applications. Drug Des. Devel. Ther. 16, 3621–3643. doi: 10.2147/DDDT.S374439 36277602 PMC9579009

[B47] Xiang Y. Yang Q. Li S. Liu Y. Li Y. Ren J. . (2025). Climate change drives northwestward migration of betula alnoides: A multi-scenario maxEnt modeling approach. Plants (Basel). 14, 2539. doi: 10.3390/plants14162539 40872161 PMC12389557

[B48] Yao W. Wang Z. Fan Y. Liu D. Ding Z. Zhou Y. . (2024). Prediction of potential habitat distributions and climate change impacts on the rare species Woonyoungia septentrionalis (Magnoliaceae) in China based on MaxEnt. Plants 14, 86. doi: 10.3390/plants14010086 39795346 PMC11723205

[B49] Záveská E. Šída O. Leong‐Škorničková J. Chumová Z. Trávníček P. Newman M. . (2024). Testing the large genome constraint hypothesis in tropical rhizomatous herbs: life strategies, plant traits and habitat preferences in gingers. Plant J. 117, 1223–1238. doi: 10.1111/tpj.16559 37991980

[B50] Zhang Y. Li J. Lin W. Qiang S. (2011). Prediction of the potential distribution of the invasive weed spring fritillary in China based on a maximum entropy ecological niche model. J. Appl. Ecol. 11, 2970–2976. 22303676

[B51] Zhang X. Zhou J. Xu H. Peng C. Meng F. (2025). Using ecological niche modelling to identify the high-quality ecological areas of Houttuynia cordata Thunb. based on secondary metabolites content. Front. Plant Sci. 16, 1641634. doi: 10.3389/fpls.2025.1641634 41164243 PMC12558978

[B52] Zhao G. Cui X. Sun J. Li T. Wang Q. Ye X. . (2021a). Analysis of the distribution pattern of Chinese Ziziphus jujuba under climate change based on optimized biomod2 and MaxEnt models. Ecol. Indic. 132, 108256. doi: 10.1016/j.ecolind.2021.10825642574925

[B53] Zhao Y. Deng X. Xiang W. Chen L. Ouyang S. (2021b). Predicting potential suitable habitats of Chinese fir under current and future climatic scenarios based on Maxent model. Ecol. Inform. 64, 101393. doi: 10.1016/j.ecoinf.2021.10139342574925

[B54] Zhong D. Li Y. Feng Y. Wang H. Zheng S. Sun X. . (2026). Spectrum-effect relationship between HPLC fingerprints and antigouty arthritis of Smilax Glabra Roxb. Biomed. Chromatogr. 40, e70448. doi: 10.1002/bmc.70448 42002404

[B55] Zhou J. Zhang X. Huo T. Xu H. Meng F. Xu N. . (2025). Predicting high-quality ecologically suitable areas of Astragalus mongholicus Bunge based on secondary metabolites content using Biomod2 model. Sci. Rep. 15, 1373. doi: 10.1038/s41598-025-85442-z 39779834 PMC11711465

