# Supplementary material for: Multi-model evaluation on integrated quality regionalization of the medicinal and edible plant Smilax glabra
Source: Front Plant Sci. 2026 Oct 14;17:1966447. doi: 10.3389/fpls.2026.1966447 (PMC13713457; doi:10.3389/fpls.2026.1966447)
Supplement: Supplementary file 1 [file DataSheet1.pdf]

Supplementary Material

1 Supplementary Figures and Tables

1.1 Supplementary Figures

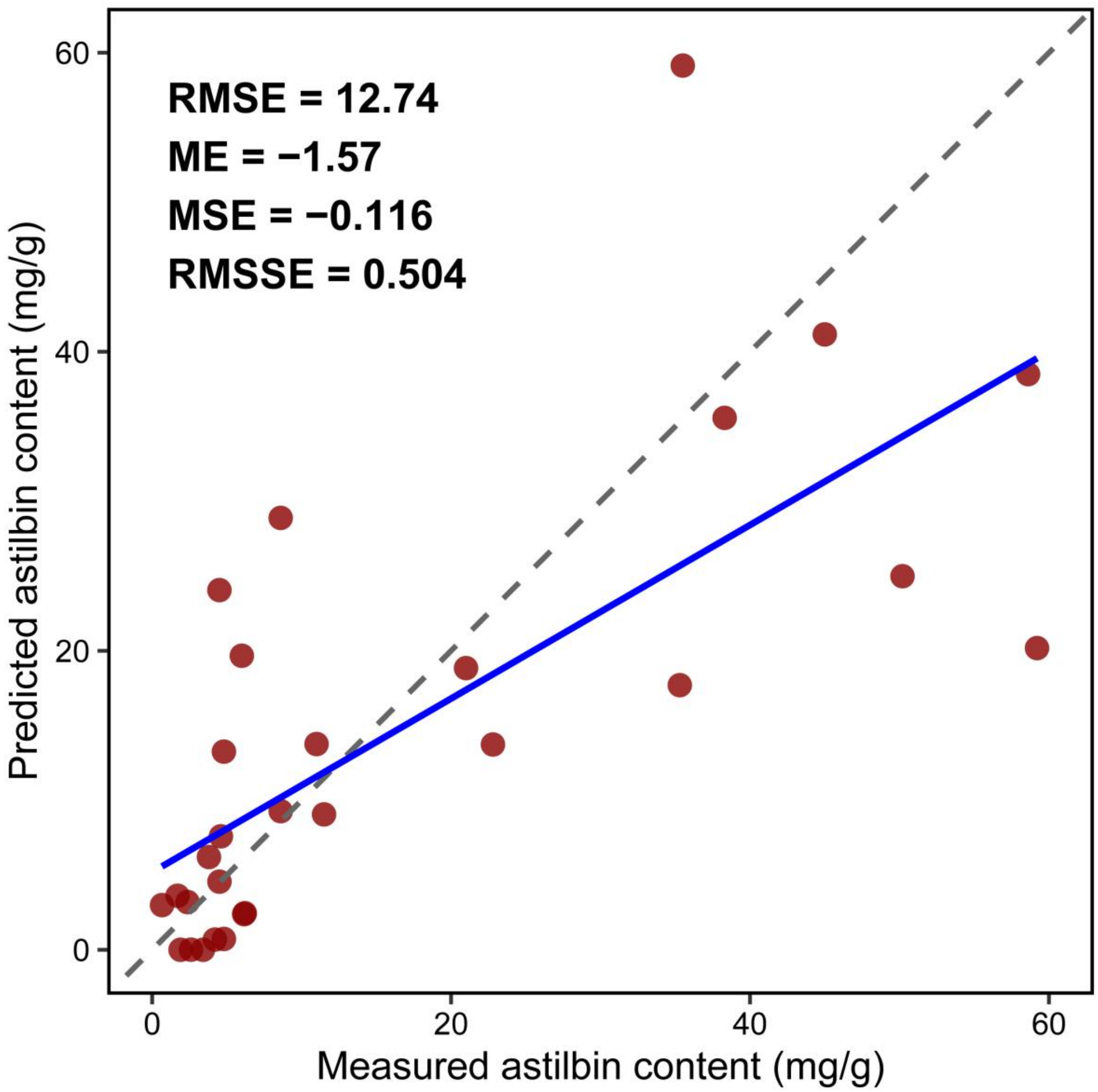

Supplementary Figure 1. Scatter plot of leave-one-out cross-validation (LOOCV) for the Universal Co-kriging interpolation model of astilbin content.

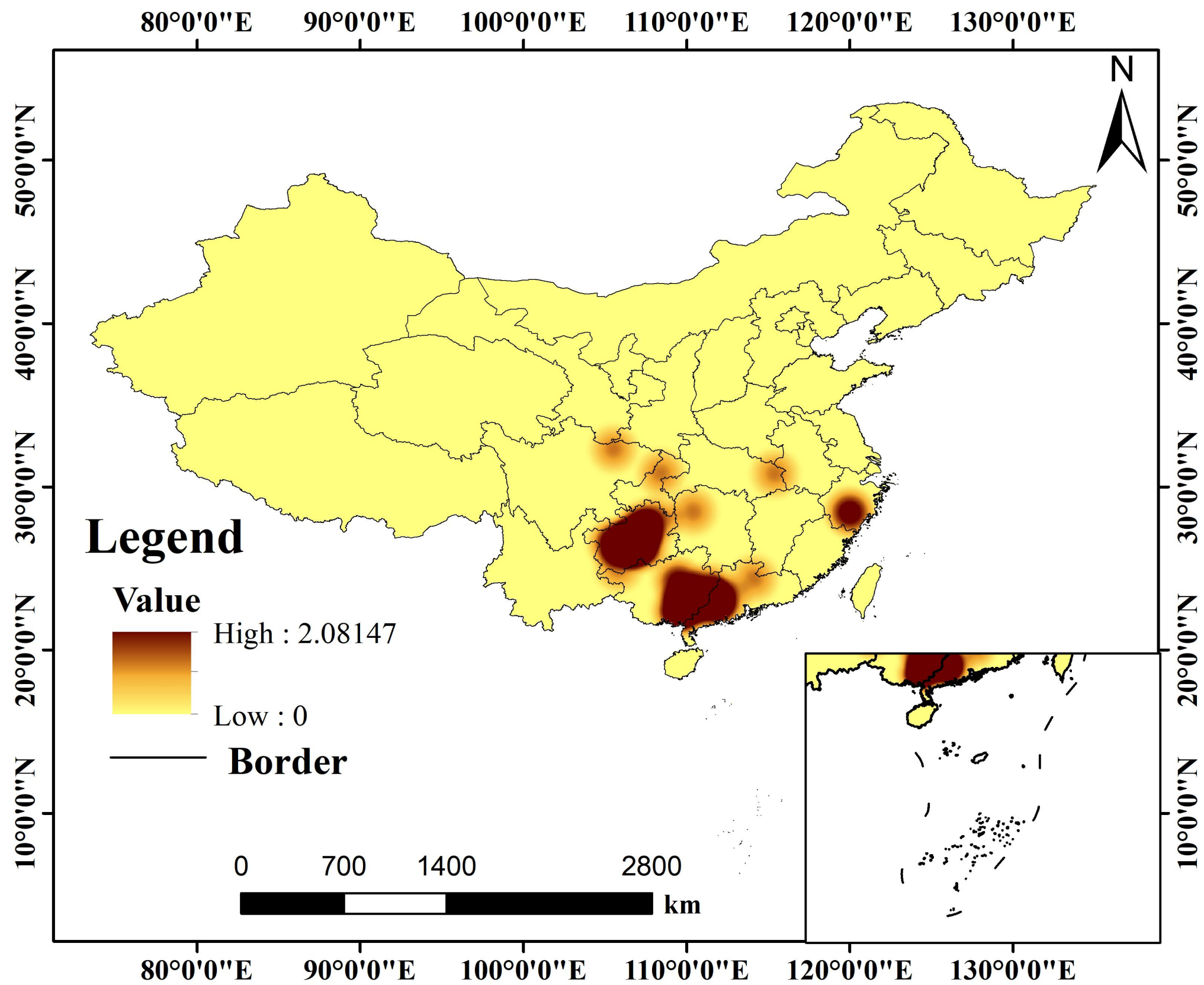

Supplementary Figure 2. Kernel density map of the sampling locations for astilbin content analysis in *Smilax glabra*.

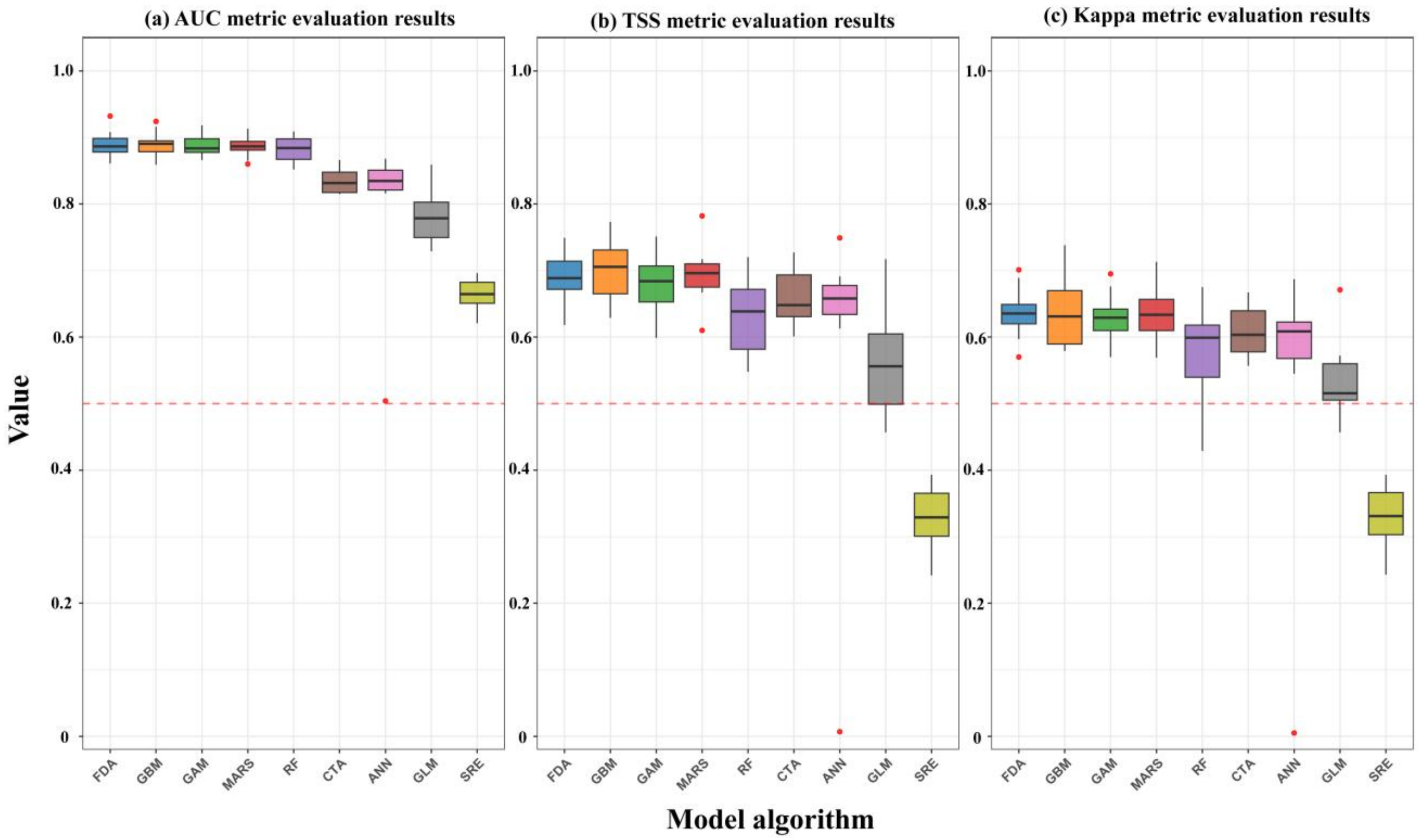

Supplementary Figure 3. Accuracy verification results for nine single-model algorithms. (a) AUC metric evaluation results; (b) TSS metric evaluation results; (c) Kappa metric evaluation results.

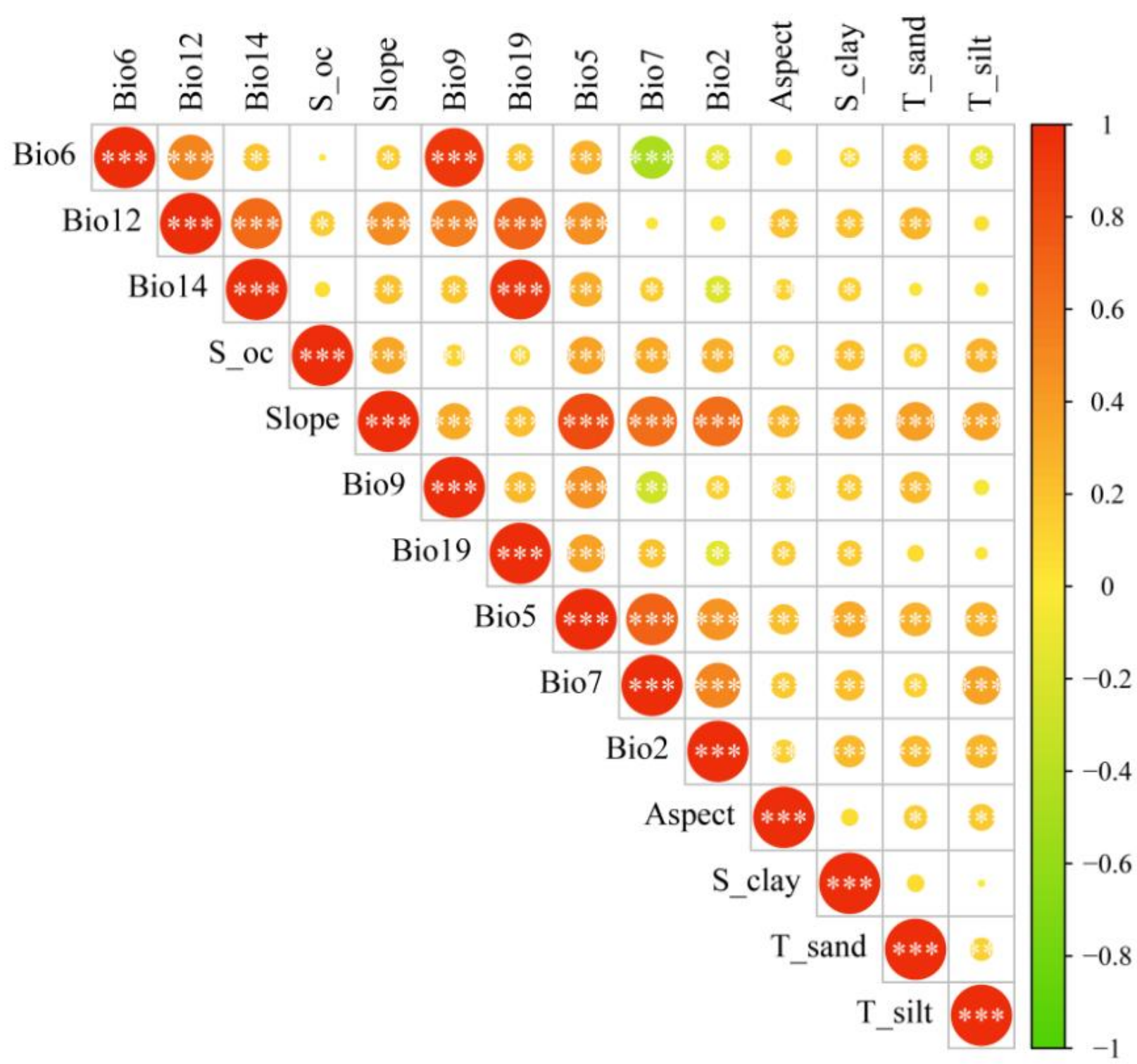

Supplementary Figure 4. Correlation Heatmap of Environmental Factors.

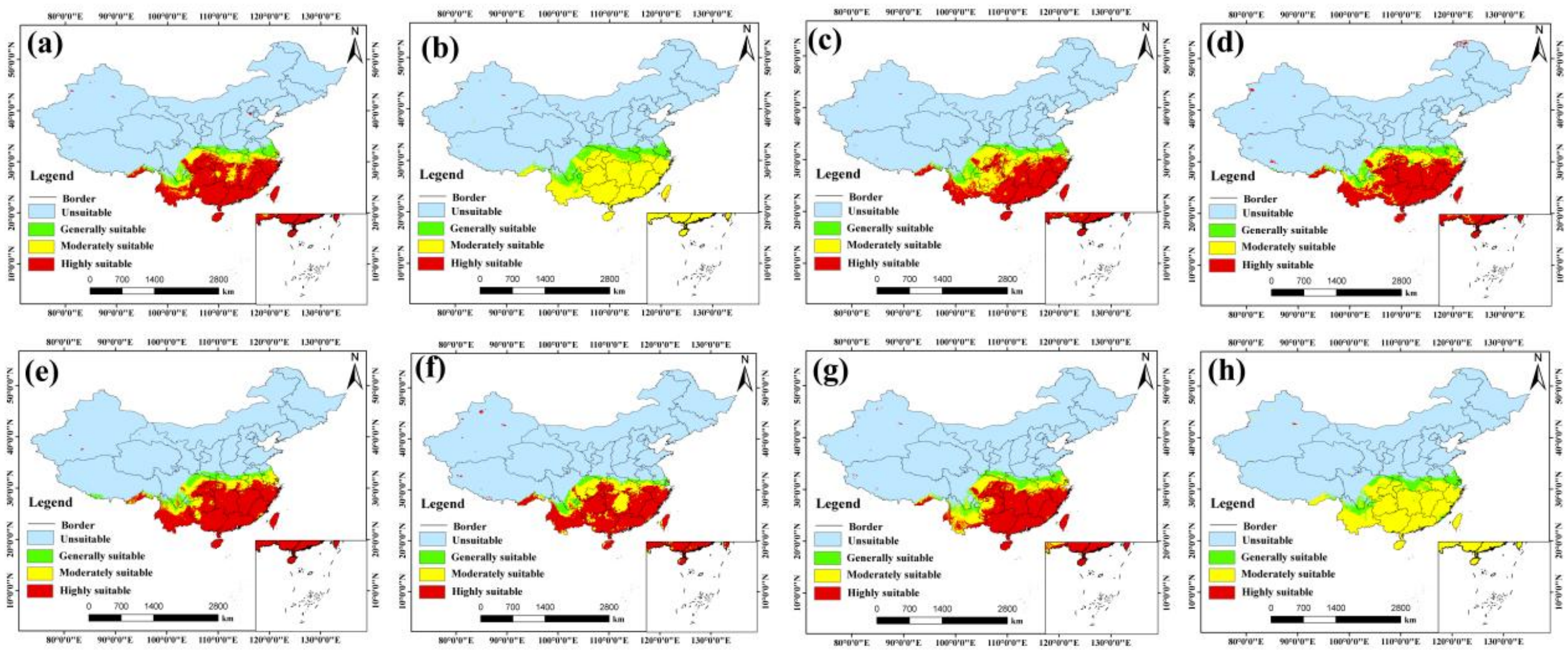

Supplementary Figure 5. Potential distribution of *Smilax glabra* under future climate conditions as modelled by GBM: (a) 2041–2060, SSP126; (b)2081–2100, SSP126; (c) 2041–2060, SSP245; (d) 2081–2100, SSP245; (e) 2041–2060, SSP370; (f) 2081–2100, SSP370; (g) 2041–2060, SSP585; (h) 2081–2100, SSP585.

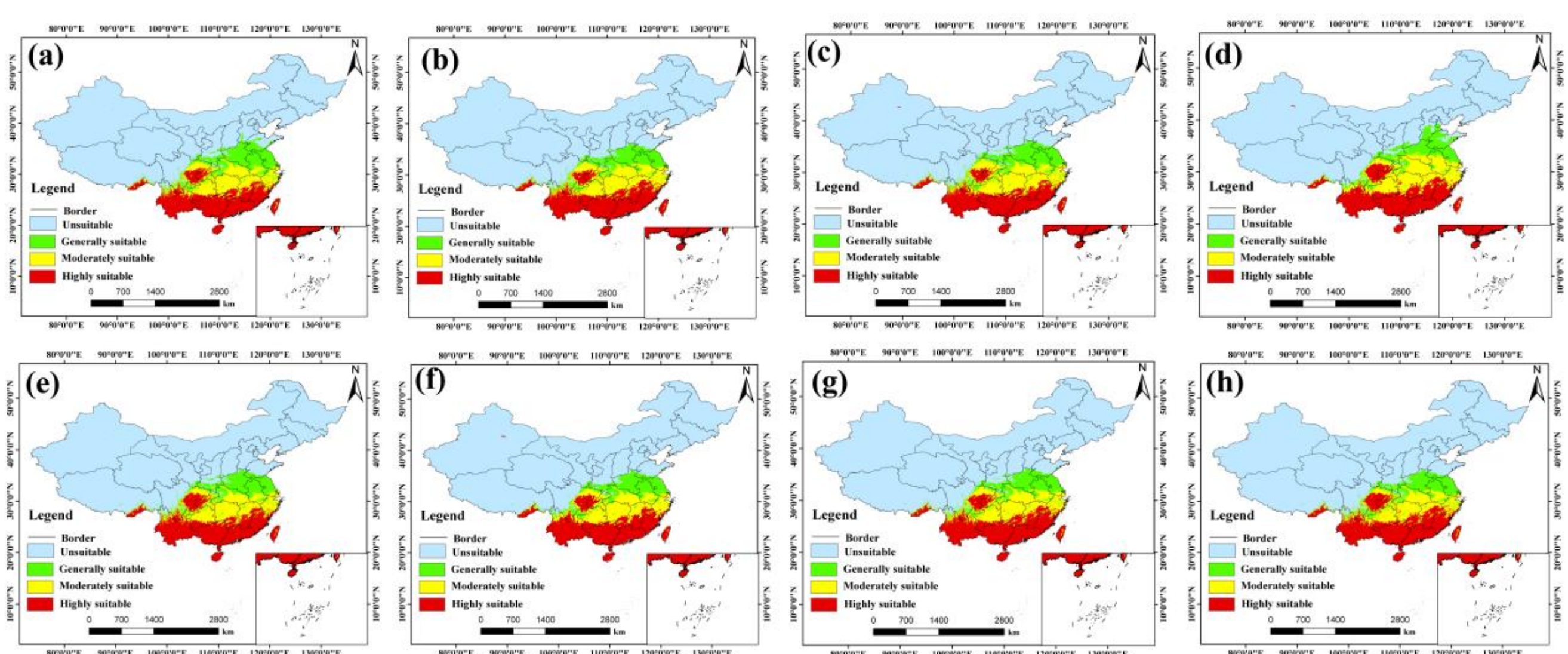

Supplementary Figure 6. Potential distribution of *Smilax glabra* under future climate conditions as modelled by FDA: (a) 2041–2060, SSP126; (b)2081–2100, SSP126; (c) 2041–2060, SSP245; (d) 2081–2100, SSP245; (e) 2041–2060, SSP370; (f) 2081–2100, SSP370; (g) 2041–2060, SSP585; (h) 2081–2100, SSP5855.

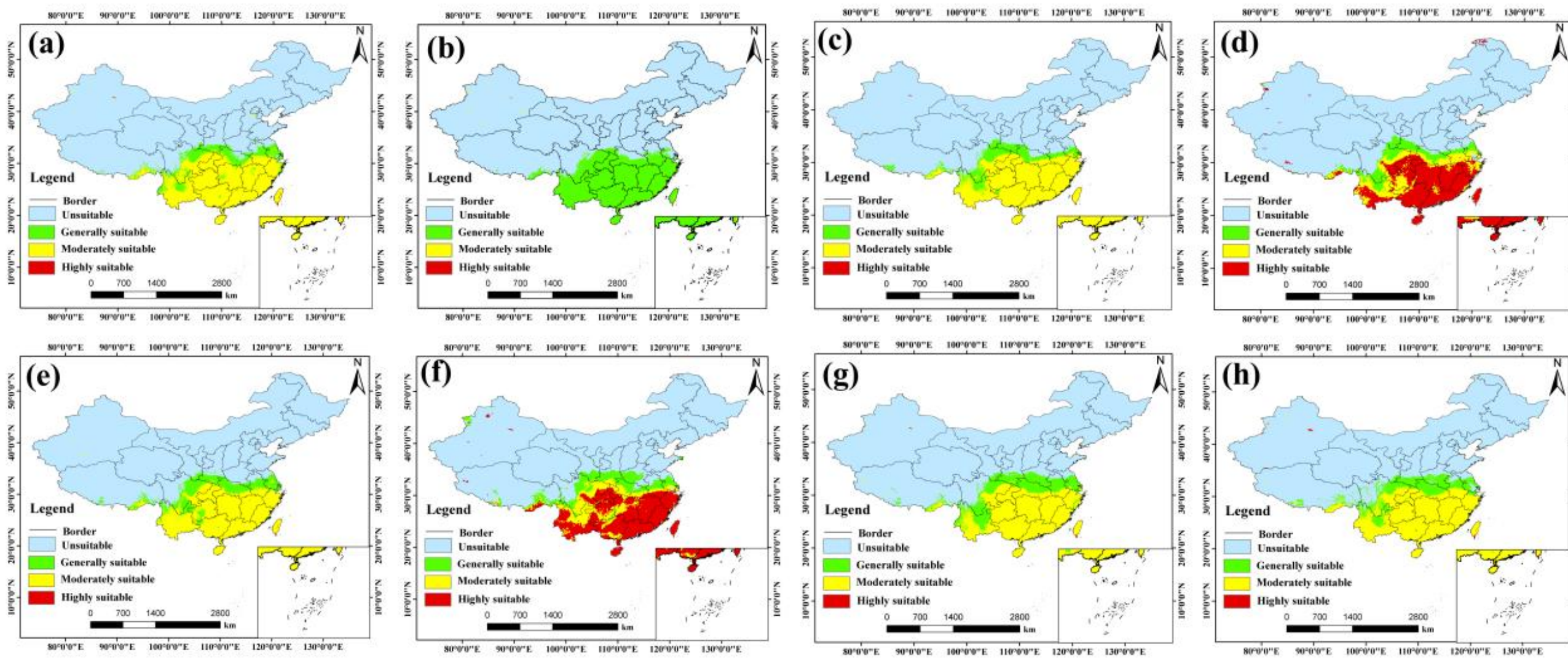

Supplementary Figure 7. Potential distribution of *Smilax glabra* under future climate conditions as modelled by GAM: (a) 2041–2060, SSP126; (b)2081–2100, SSP126; (c) 2041–2060, SSP245; (d) 2081–2100, SSP245; (e) 2041–2060, SSP370; (f) 2081–2100, SSP370; (g) 2041–2060, SSP585; (h) 2081–2100, SSP585.

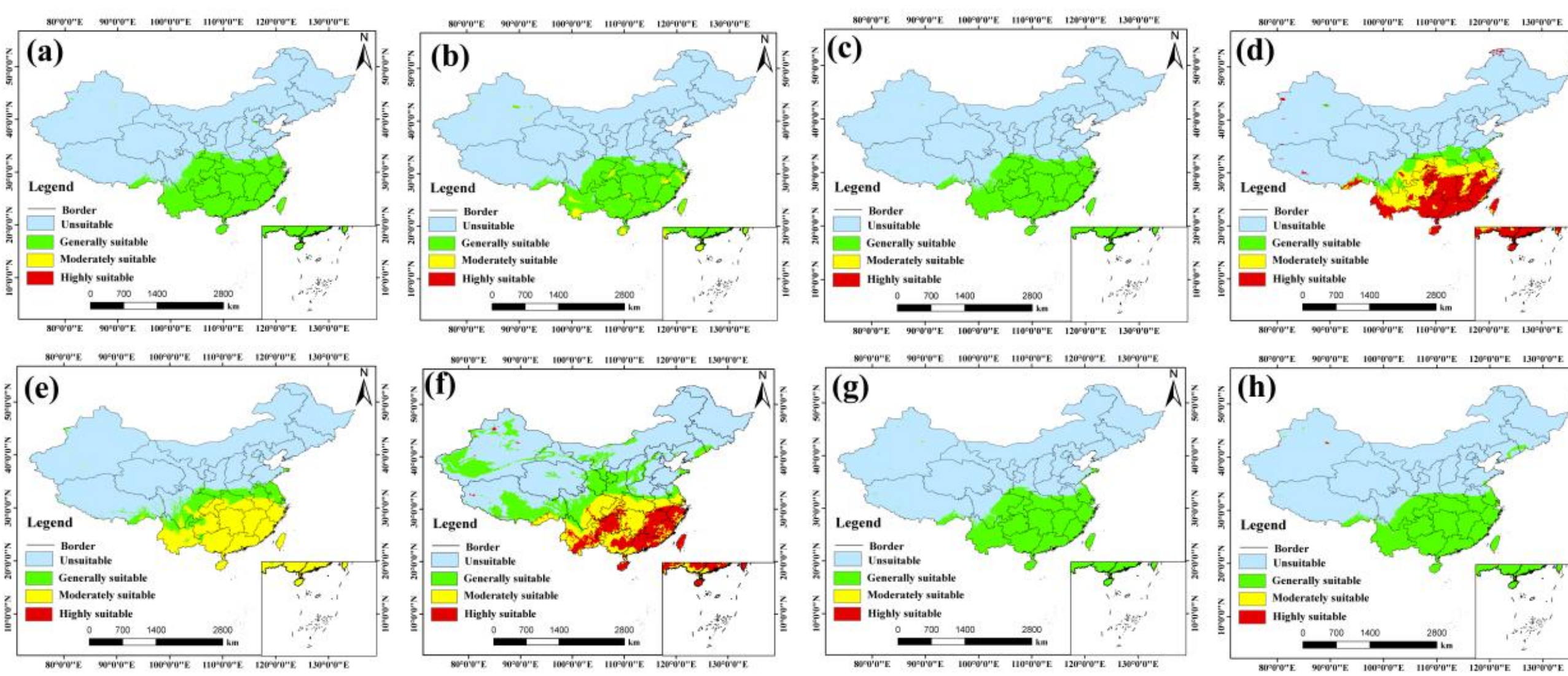

Supplementary Figure 8. Potential distribution of *Smilax glabra* under future climate conditions as modelled by MARS: (a) 2041–2060, SSP126; (b)2081–2100, SSP126; (c) 2041–2060, SSP245; (d) 2081–2100, SSP245; (e) 2041–2060, SSP370; (f) 2081–2100, SSP370; (g) 2041–2060, SSP585; (h) 2081–2100, SSP585.

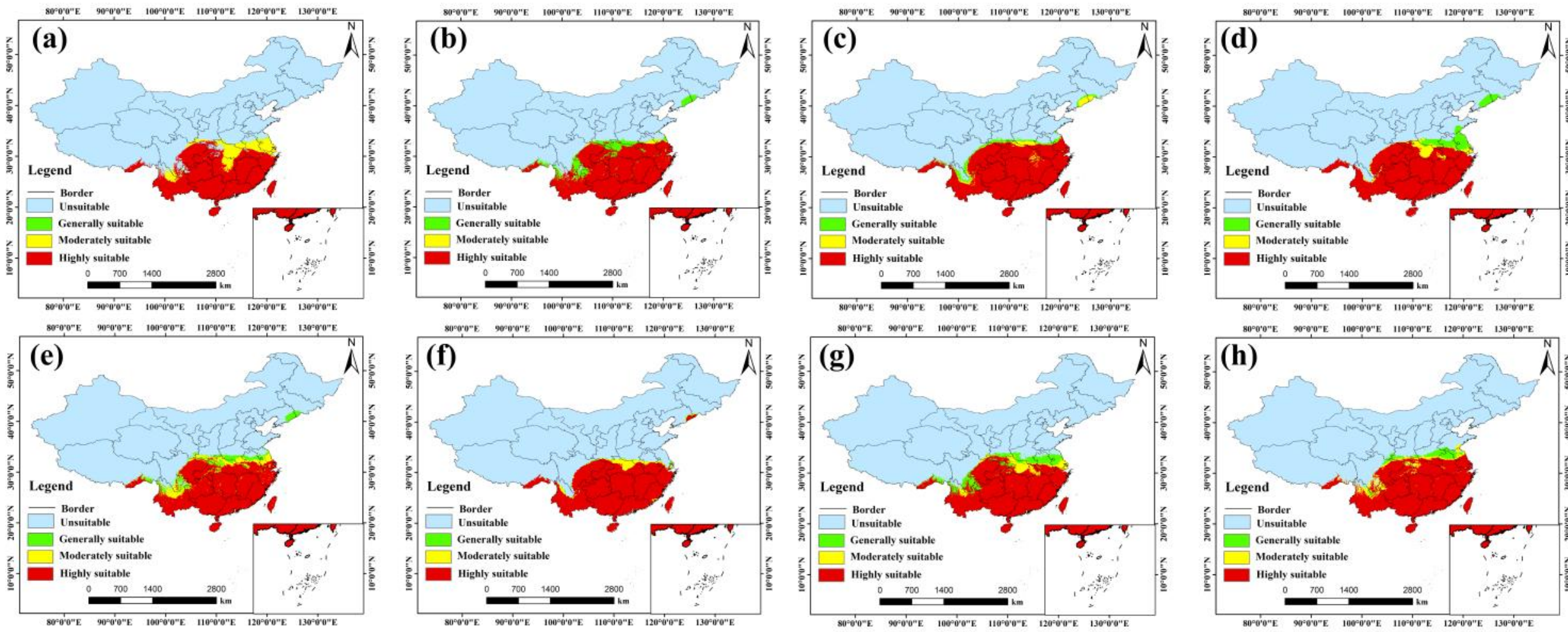

Supplementary Figure 9. Potential distribution of *Smilax glabra* under future climate conditions as modelled by CTA: (a) 2041–2060, SSP126; (b)2081–2100, SSP126; (c) 2041–2060, SSP245; (d) 2081–2100, SSP245; (e) 2041–2060, SSP370; (f) 2081–2100, SSP370; (g) 2041–2060, SSP585; (h) 2081–2100, SSP585.

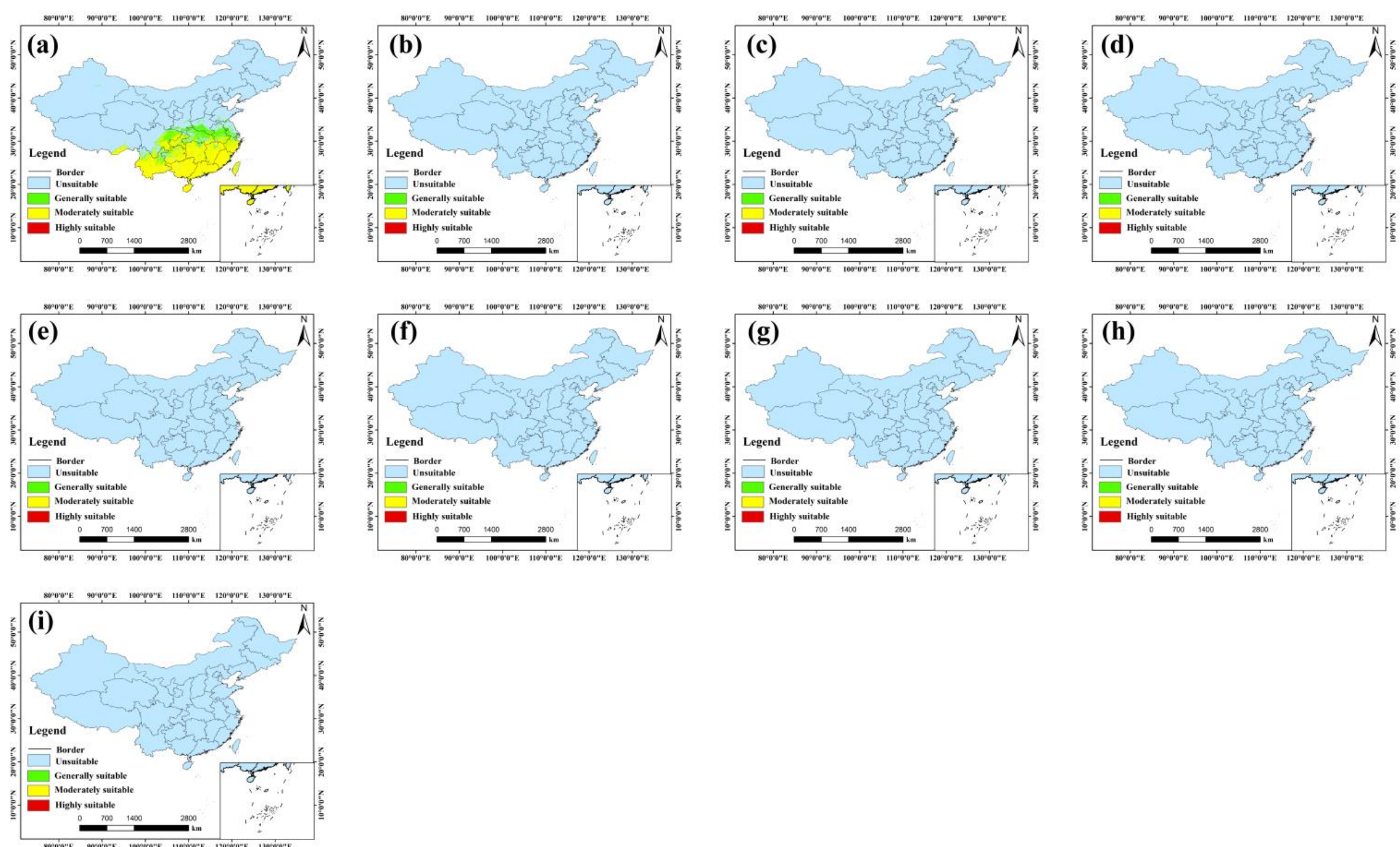

Supplementary Figure 10. Potential distribution of *Smilax glabra* under current and future climate conditions projected by the Ensemble model based on a fixed threshold strategy：(a) Current period; (b) 2041–2060, SSP126; (c) 2081–2100, SSP126; (d) 2041–2060, SSP245; (e) 2081–2100, SSP245; (f) 2041–2060, SSP370; (g) 2081–2100, SSP370; (h) 2041–2060, SSP585; (i) 2081–2100, SSP585.(Note: The threshold defining suitability classes was fixed across all scenarios based on the Maximum Training Sensitivity plus Specificity of the current climate model).

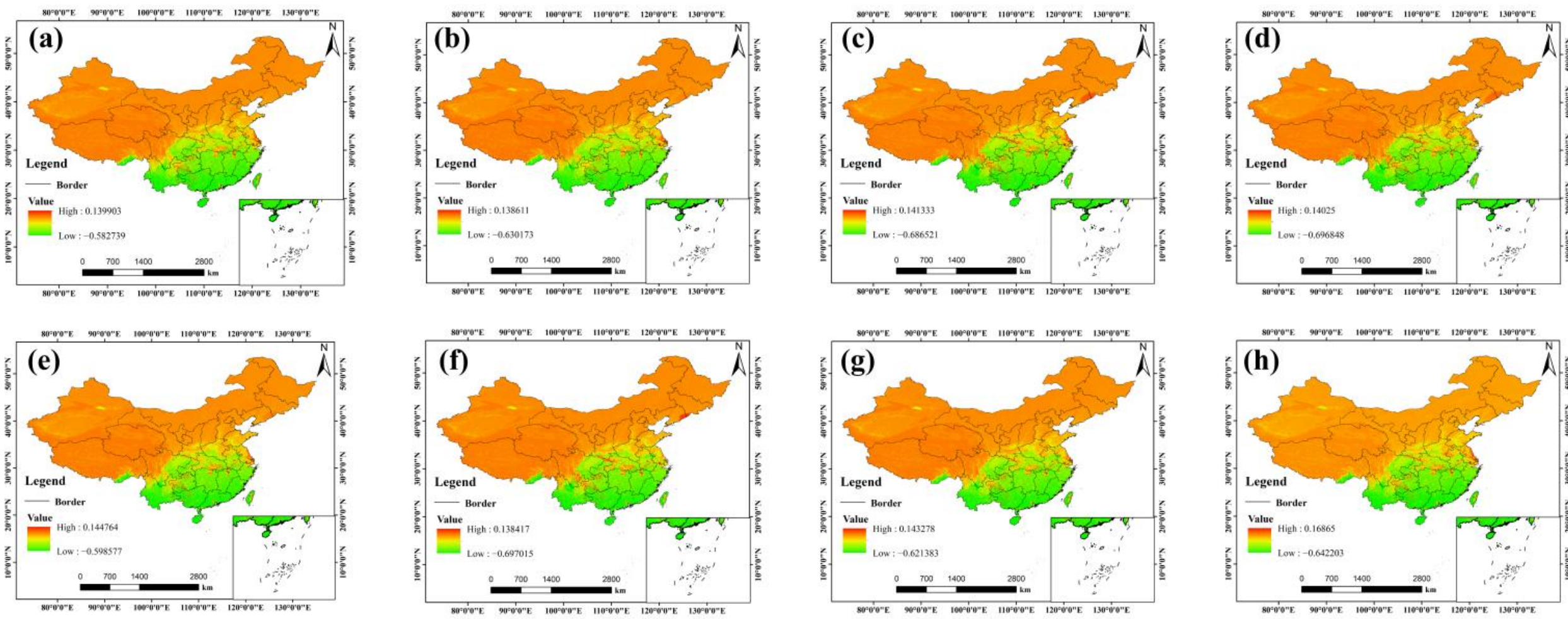

Supplementary Figure 11. Spatial changes in continuous suitability probabilities of *Smilax glabra* under future climate conditions relative to the current period (Delta Maps generated by the Ensemble model). Positive values indicate habitat improvement, while negative values indicate habitat degradation. (a) 2041–2060, SSP126; (b) 2081–2100, SSP126; (c) 2041–2060, SSP245; (d) 2081–2100, SSP245; (e) 2041–2060, SSP370; (f) 2081–2100, SSP370; (g) 2041–2060, SSP585; (h) 2081–2100, SSP585.

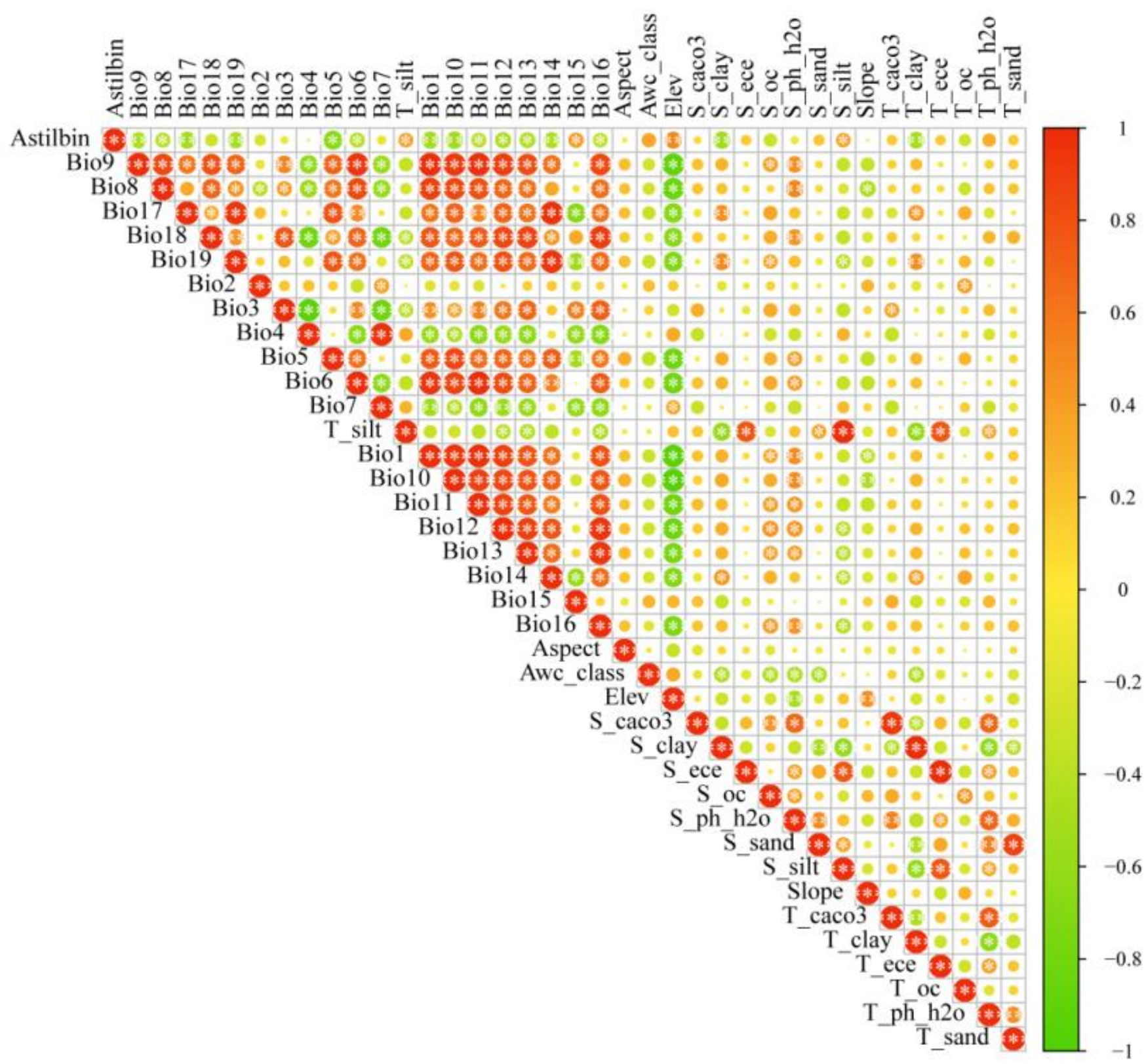

Supplementary Figure 12. Results of Spearman's correlation analysis for component-relatedness.

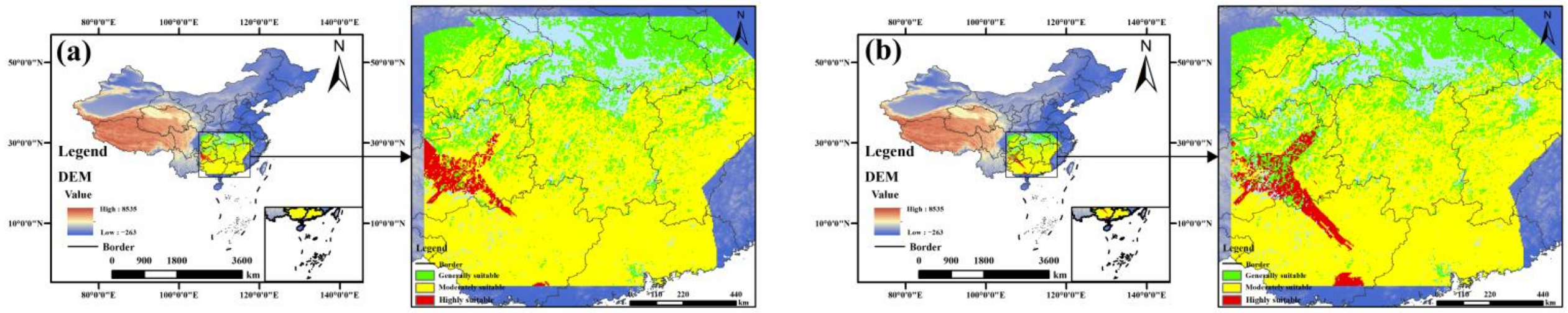

Supplementary Figure 13. Integrated quality regions of *Smilax glabra.* under different weighting schemes: (a) Ecological vs. Chemical = 0.3:0.7; (b) Ecological vs. Chemical = 0.7:0.3.

1.2 Supplementary Tables

Supplementary Table 1. Composition data of active components in *Smilax glabra* from different geographical origins.

**Sample**	**Geographic Origin**	**Latitude (°N)**	**Longitude (°E)**	**Astilbin Content**	**Unit**	**Extraction Method**	**Analytical Method**	**Original Source**	**Collection Time**
S1	Liujiang District, Liuzhou City, Guangxi	24.261957	109.332811	4.5	mg/g	Ultrasonication (60% Methanol)	HPLC-UV (291 nm)	Bai et al. (2013)	2009
S2	Xiangzhou County, Laibin City, Guangxi	23.979915	109.711314	4.6	mg/g	Ultrasonication (60% Methanol)	HPLC-UV (291 nm)	Bai et al. (2013)	2009
S3	Wanzhou District, Chongqing City	30.813418	108.415328	4.2	mg/g	Ultrasonication (60% Methanol)	HPLC-UV (291 nm)	Bai et al. (2013)	2009
S4	Luotian County, Huanggang City, Hubei	30.78886	115.405696	3.4	mg/g	Ultrasonication (60% Methanol)	HPLC-UV (291 nm)	Bai et al. (2013)	2009
S5	Gaoyao District, Zhaoqing City, Guangdong	23.030935	112.464501	0.66	mg/g	Ultrasonication (70% Ethanol)	HPLC-UV (291 nm)	Zhang et al. (2010)	2010
S6	Jiange County, Guangyuan City, Sichuan	32.294061	105.53134	5.5	mg/g	Ultrasonication (70% Ethanol)	HPLC-UV (291 nm)	Zhang et al. (2010)	2010
S7	Liandu District, Lishui City, Zhejiang	28.470564	119.907542	4.179	mg/g	Ultrasonication (Methanol)	HPLC-UV (291 nm)	Xu et al. (2015)	2011
S8	Qingtian County, Lishui City, Zhejiang	28.349955	120.100487	4.2918	mg/g	Ultrasonication (Methanol)	HPLC-UV (291 nm)	Xu et al. (2015)	2011
S9	Yunan County, Yunfu City, Guangdong	22.924989	111.68048	2.367	mg/g	Ultrasonication (Methanol)	HPLC-UV (291 nm)	Xu et al. (2015)	2011
S10	Yunan County, Yunfu City,	22.685654	111.505593	6.155	mg/g	Ultrasonication	HPLC-UV (291 nm)	Xu et al. (2015)	2011

	Guangdong					(Methanol)			
S11	Yunan County, Yunfu City, Guangdong	22.9419 6	111.68204 3	1.702	mg/g	Ultrasonicati on (Methanol)	HPLC-UV (291 nm)	Xu et al. (2015)	2011
S12	Cangwu County, Wuzhou City, Guangxi	23.5932 49	110.96674 6	4.5025	mg/g	Ultrasonicati on (Methanol)	HPLC-UV (291 nm)	Xu et al. (2015)	2011
S13	Wengyuan County, Shaoguan City, Guangdong	24.3568 76	114.13641 4	2.6	mg/g	Ultrasonicati on (60% Methanol)	HPLC-UV (291 nm)	Li et al. (2007)	2005
S14	Yuanling County, Huaihua City, Hunan	28.4584 2	110.40021 3	4.8	mg/g	Ultrasonicati on (60% Methanol)	HPLC-UV (291 nm)	Li et al. (2007)	2005
S15	Xingye County, Yulin City, Guangxi	22.7421 65	109.88177 4	11.5	mg/g	Ultrasonicati on (70% Ethanol)	HPLC-UV (291 nm)	Chen et al. (2011)	2011
S16	Bobai County, Yulin City, Guangxi	22.2792 14	109.98240 9	8.6	mg/g	Ultrasonicati on (70% Ethanol)	HPLC-UV (291 nm)	Chen et al. (2011)	2011
S17	Cangwu County, Wuzhou City, Guangxi	23.8739 92	111.55428 9	6.2	mg/g	Ultrasonicati on (70% Ethanol)	HPLC-UV (291 nm)	Chen et al. (2011)	2011
S18	Pubei County, Qinzhou City, Guangxi	22.2782 22	109.56265 5	22.8	mg/g	Ultrasonicati on (70% Ethanol)	HPLC-UV (291 nm)	Chen et al. (2011)	2011
S19	Lingshan County, Qinzhou City, Guangxi	22.4226 79	109.29717 7	11	mg/g	Ultrasonicati on (70% Ethanol)	HPLC-UV (291 nm)	Chen et al. (2011)	2011
S20	Huaxi District, Guiyang City, Guizhou	26.5226 57	106.59739 8	58.6	mg/g	Ultrasonicati on (70% Ethanol)	HPLC-UV (291 nm)	He et al. (2012)	2012
S21	Huaxi District, Guiyang City, Guizhou	26.3846 84	106.68828 7	38.3	mg/g	Ultrasonicati on (70% Ethanol)	HPLC-UV (291 nm)	He et al. (2012)	2012

S22	Longli County, Guizhou	26.441054	106.924436	59.2	mg/g	Ultrasonication (70% Ethanol)	HPLC-UV (291 nm)	He et al. (2012)	2012
S23	Longli County, Guizhou	26.233386	107.038593	35.3	mg/g	Ultrasonication (70% Ethanol)	HPLC-UV (291 nm)	He et al. (2012)	2012
S24	Qingzhen City, Guiyang City, Guizhou	26.584471	106.19782	45	mg/g	Ultrasonication (70% Ethanol)	HPLC-UV (291 nm)	He et al. (2012)	2012
S25	Zhijin County, Bijie City, Guizhou	26.530008	105.611029	35.5	mg/g	Ultrasonication (70% Ethanol)	HPLC-UV (291 nm)	He et al. (2012)	2012
S26	Duyun City, Guizhou	26.274956	107.503228	21	mg/g	Ultrasonication (70% Ethanol)	HPLC-UV (291 nm)	He et al. (2012)	2012
S27	Zhijin County, Bijie City, Guizhou	26.495079	105.37669	50.2	mg/g	Ultrasonication (70% Ethanol)	HPLC-UV (291 nm)	He et al. (2012)	2012
S28	Ceheng County, Guizhou	24.989631	105.818144	1.9	mg/g	Ultrasonication (70% Ethanol)	HPLC-UV (291 nm)	He et al. (2012)	2012
S29	Kaiyang County, Guiyang City, Guizhou	27.265343	107.019193	8.6	mg/g	Ultrasonication (70% Ethanol)	HPLC-UV (291 nm)	He et al. (2012)	2012
S30	Kaiyang County, Guiyang City, Guizhou	27.286649	106.99818	4.8	mg/g	Ultrasonication (70% Ethanol)	HPLC-UV (291 nm)	He et al. (2012)	2012

Supplementary Table 2. Suitable area of *Smilax glabra* under different future climate scenarios and periods.

Model	Periods	Climate scenarios	Generally suitable area (× 10^4 km^2)	Moderately suitable area (× 10^4 km^2)	Highly suitable area (× 10^4 km^2)	Total suitable area (× 10^4 km^2)
Ensemble Model	Current	—	52.98	53.61	133.48	240.07
	2050s	SSP126	12.73	37.40	172.59	222.72
		SSP245	9.45	8.72	211.64	229.81
		SSP370	25.29	23.83	179.39	228.51
		SSP585	31.55	16.87	183.54	231.96
	2090s	SSP126	11.02	22.78	192.30	226.10
		SSP245	24.98	13.68	190.14	228.80
		SSP370	1.28	1.17	203.45	205.90
		SSP585	19.08	10.10	218.23	247.41
MaxEnt	Current	—	54.03	66.43	89.70	210.16
	2050s	SSP126	54.74	76.33	88.00	219.07
		SSP245	50.50	77.74	89.69	217.92
		SSP370	53.66	68.11	102.35	224.12
		SSP585	55.47	67.93	97.87	221.27
	2090s	SSP126	55.16	78.63	86.70	220.49
		SSP245	64.91	75.13	78.88	218.92
		SSP370	46.77	89.52	67.66	203.96

		SSP585	47.40	75.36	91.49	214.25
GBM	Current	—	52.61	65.66	121.73	240.00
	2050s	SSP126	27.88	42.63	145.24	215.74
		SSP245	28.23	64.75	121.37	214.35
		SSP370	26.97	42.32	151.56	220.85
		SSP585	29.88	52.21	131.34	213.43
	2090s	SSP126	50.36	164.01	1.16	215.52
		SSP245	31.28	44.73	142.87	218.88
		SSP370	20.47	50.14	135.78	206.40
		SSP585	38.29	169.76	0.49	208.53
FDA	Current	—	52.25	50.55	117.58	220.38
	2050s	SSP126	68.71	75.11	106.10	249.93
		SSP245	64.63194	79.59202	102.3333	246.56
		SSP370	60.91	74.63	110.48	246.02
		SSP585	58.61	76.16	110.41	245.17
	2090s	SSP126	58.34	77.34	109.89	245.58
		SSP245	60.99	87.09	112.81	260.89
		SSP370	58.11	75.85	107.10	241.06
		SSP585	58.23264	70.18924	114.5781	243.00
GAM	Current	—	57.13	71.97	126.97	256.07
	2050s	SSP126	49.32	177.43	0.48	227.24

		SSP245	50.81	170.08	0.51	221.40
		SSP370	61.57	168.10	0.27	229.94
		SSP585	73.93	164.74	0.41	239.08
	2090s	SSP126	200.39	1.39	0.21	201.99
		SSP245	44.41	59.31	122.95	226.68
		SSP370	58.61	61.75	134.49	254.86
		SSP585	67.23	174.44	1.01	242.68
MARS	Current	—	302.14	87.64	147.27	537.05
	2050s	SSP126	221.63	0.43	0.09	222.15
		SSP245	214.30	0.30	0.24	214.85
		SSP370	77.03	168.43	0.12	245.58
		SSP585	222.31	0.35	0.09	222.76
	2090s	SSP126	197.94	13.42	0.18	211.54
		SSP245	48.51	89.39	106.13	244.02
		SSP370	181.51	118.33	88.45	388.29
		SSP585	224.74	0.66	0.46	225.86
CTA	Current	—	37.44	202.37	0.00	239.81
	2050s	SSP126	0.89	36.51	172.59	209.99
		SSP245	14.80	12.14	202.87	229.81
		SSP370	23.63	24.84	180.12	228.59
		SSP585	31.54	16.87	183.54	231.95

	2090s	SSP126	29.35	4.76	189.36	223.47
		SSP245	24.98	13.68	190.14	228.80
		SSP370	2.45	11.61	191.84	205.90
		SSP585	14.76	18.38	201.40	234.53

Supplementary Table 3. Centroid migration coordinates and distances of *Smilax glabra.* predicted by single algorithms and the ensemble model under different future climate scenarios.

Model	Periods	Climate scenarios	Longitude (° E)	Latitude (° N)	Migration distance (km)
Ensemble Model	Current	—	109.80	29.85	—
	2050s	SSP126	105.61	28.87	420.80
		SSP245	103.32	29.70	625.75
		SSP370	105.46	29.03	431.00
		SSP585	106.71	29.36	304.42
	2090s	SSP126	106.28	29.08	68.64
		SSP245	106.66	29.29	326.32
		SSP370	109.82	30.46	450.77
		SSP585	105.00	29.89	175.57
MaxEnt	Current	—	109.72	27.07	—
	2050s	SSP126	106.21	27.06	347.46
		SSP245	106.04	26.97	364.67
		SSP370	104.62	27.61	506.72
		SSP585	105.40	27.39	428.47
	2090s	SSP126	105.04	27.12	115.71
		SSP245	103.83	26.71	220.82
		SSP370	105.78	26.47	170.70
		SSP585	104.72	27.37	66.92

GBM	Current	—	108.20	29.98	—
	2050s	SSP126	105.49	28.80	293.02
		SSP245	105.44	28.73	301.76
		SSP370	105.32	29.15	293.88
		SSP585	104.74	28.41	378.22
	2090s	SSP126	104.99	29.79	119.67
		SSP245	103.82	29.49	177.93
		SSP370	103.99	28.60	143.21
		SSP585	105.34	29.91	176.12
FDA	Current	—	110.65	29.61	—
	2050s	SSP126	105.15	28.36	552.71
		SSP245	105.30	28.63	531.15
		SSP370	104.86	28.56	574.99
		SSP585	104.63	28.44	600.45
	2090s	SSP126	104.62	28.40	52.83
		SSP245	105.50	28.59	19.79
		SSP370	105.17	28.63	31.04
		SSP585	104.72	28.35	594.24
GAM	Current	—	108.48	32.26	—
	2050s	SSP126	108.08	29.56	302.45
		SSP245	103.38	29.53	573.18

		SSP370	107.94	29.63	296.92
		SSP585	102.87	29.43	620.31
	2090s	SSP126	107.87	31.32	196.73
		SSP245	103.53	29.29	30.60
		SSP370	100.67	30.11	702.14
		SSP585	104.29	30.68	194.91
MARS	Current	—	104.13	34.32	—
	2050s	SSP126	104.99	31.47	326.41
		SSP245	99.09	33.00	488.14
		SSP370	104.43	30.02	477.97
		SSP585	103.18	31.96	276.40
	2090s	SSP126	105.87	28.76	313.69
		SSP245	103.01	29.34	551.13
		SSP370	100.84	32.05	409.05
		SSP585	102.77	33.56	181.42
CTA	Current	—	112.13	32.15	—
	2050s	SSP126	108.82	29.64	422.05
		SSP245	103.49	28.97	899.57
		SSP370	105.40	29.02	732.41
		SSP585	106.72	29.36	603.53
	2090s	SSP126	106.97	28.73	624.38

		SSP245	106.66	29.29	612.21
		SSP370	109.31	30.46	327.61
		SSP585	104.36	28.53	847.34

Supplementary Table 4. Spearman correlation coefficients between astilbin content and environmental variables.

Variable	Description	Correlation Coefficient (r)	Original P-value	FDR-adjusted P-value (q-value)
Bio5	Maximum temperature of the warmest month	– 0.641**	0.0001	0.0029
Bio10	Mean temperature of the warmest quarter	– 0.529*	0.0019	0.0214
S_clay	Subsoil clay content	– 0.526*	0.002	0.0214
Bio1	Annual mean temperature	– 0.513*	0.0027	0.0214
Bio17	Precipitation of the driest quarter	– 0.508*	0.003	0.0214
Elev	Elevation	0.501*	0.0035	0.0214
T_clay	Topsoil clay content	– 0.487*	0.0047	0.0248
Bio19	Precipitation of the coldest quarter	– 0.460*	0.0081	0.0339
Bio14	Precipitation of the driest month	– 0.456*	0.0087	0.0339
Bio9	Mean temperature of the driest quarter	– 0.453*	0.0092	0.0339
Bio12	Annual precipitation	– 0.447*	0.0104	0.035

Bio13	Precipitation of the wettest month	– 0.435*	0.0129	0.0396
Bio6	Minimum temperature of the coldest month	– 0.396	0.0247	0.0606
Bio8	Mean temperature of the wettest quarter	– 0.393	0.0262	0.0606
Bio11	Mean temperature of the coldest quarter	– 0.382	0.031	0.0675
Bio16	Precipitation of the wettest quarter	– 0.366	0.0393	0.0766
Bio15	Precipitation seasonality	0.4	0.0232	0.0606
T_ph_h2o	Topsoil pH	0.407	0.0229	0.0606
T_silt	Topsoil silt content	0.368	0.038	0.0766
S_silt	Subsoil silt content	0.362	0.0416	0.077
Awc_class	Soil available water content	0.349	0.0506	0.0891

Notes: Statistical significance was re-evaluated after applying the Benjamini-Hochberg False Discovery Rate (FDR) correction to account for multiple testing across 37 environmental variables.

Correlation is significant at the 0.01 level based on the FDR-adjusted P-value ($q < 0.01$, two-tailed).

* Correlation is significant at the 0.05 level based on the FDR-adjusted P-value ($q < 0.05$, two-tailed). Variables lacking an asterisk lost their statistical significance after FDR correction and are interpreted as potential false-positive associations.
